# COF Scaffold Membrane with Gate-Lane Nanostructure for Efficient Li^+^/Mg^2+^ Separation

**DOI:** 10.1007/s40820-025-01972-1

**Published:** 2026-01-02

**Authors:** Zixuan Zhang, Yan Kong, Runlai Li, Xiaolin Yue, Hao Deng, Yu Zheng, Sui Zhang, Runnan Zhang, Zhongyi Jiang

**Affiliations:** 1 Joint School of National University of Singapore and Tianjin University, International Campus of Tianjin University, Binhai New City, Fuzhou, 350207 People’s Republic of China; 2 Department of Chemical and Biomolecular Engineering, National University of Singapore, 4 Engineering Drive 4, Singapore, 117585 Singapore; 3 Key Laboratory for Green Chemical Technology of Ministry of Education, School of Chemical Engineering and Technology, Tianjin University, Tianjin, 300072 People’s Republic of China; 4 Ningbo Key Laboratory of Green Petrochemical Carbon Emission Reduction Technology and Equipment, Zhejiang Institute of Tianjin University, Ningbo, 315201 Zhejiang People’s Republic of China; 5 College of Polymer Science and Engineering, State Key Laboratory of Polymer Materials Engineering, Sichuan University, Chengdu, 610065 People’s Republic of China; 6 State Key Laboratory of Synthetic Biology, Tianjin University, Tianjin, 300072 People’s Republic of China

**Keywords:** Covalent organic framework, Polyethyleneimine, Scaffold membrane, Lithium/magnesium nanofiltration separation, Ion mixing effect

## Abstract

Due to complex ion–ion and ion–membrane interactions, creating innovative membrane structures to acquire favorable ion mixing effect and high separation performance remains a big challenge. Herein, we design covalent organic framework (COF) scaffold membrane with gate-lane nanostructure for efficient Li^+^/Mg^2+^ separation. COF nanosheets, serving as the scaffold, are intercalated by polyethyleneimine (PEI) to form the permeating layer. Subsequently, PEI on the surface reacts with 1,4-phenylene diisocyanate to form the polyurea gating layer. The gating layer, bearing tailored smaller pore size, affords high rejection to co-ions (Mg^2+^) and thus high Li^+^/Mg^2+^ selectivity. The permeating layer, with asymmetric charge and spatial nanostructure for creating individual lanes of Li^+^ and Cl^−^, facilitates Li^+^ transport and thus high Li^+^ permeability. The optimum COF scaffold membrane exhibits the permeance of 11.5 L m^−2^ h^−1^/bar^−1^ and true selectivity of 231.9 with Li^+^ enrichment of 120.2% at the Mg^2+^/Li^+^ mass ratio of 50, exceeding the ideal selectivity of 80.5 and outperforming all ever-reported positively charged nanofiltration membranes. Our work may stimulate the further thinking about how to design the hierarchical membrane structure to achieve favorable ion mixing effect and break the membrane permeability–selectivity trade-off in chemical separations.

**Supplementary Information:**

The online version contains supplementary material available at 10.1007/s40820-025-01972-1.

## Highlights

- Covalent organic framework (COF) scaffold membranes with gate-lane nanostructure were prepared.
- The gating layer affords high rejection to Mg^2+^ and thus high Li^+^/Mg^2+^ selectivity. The permeating layer bearing Li^+^ lanes and Cl^−^ lanes facilitates Li^+^ transport and thus high Li^+^ permeability.
- The COF scaffold membrane exhibits the true selectivity of 231.9 with Li^+^ enrichment of 120.2% at the Mg^2+^/Li^+^ mass ratio of 50, exceeding the ideal selectivity of 80.5.

**Supplementary Information:**

The online version contains supplementary material available at 10.1007/s40820-025-01972-1.

## Graphical abstract

**Figure:**
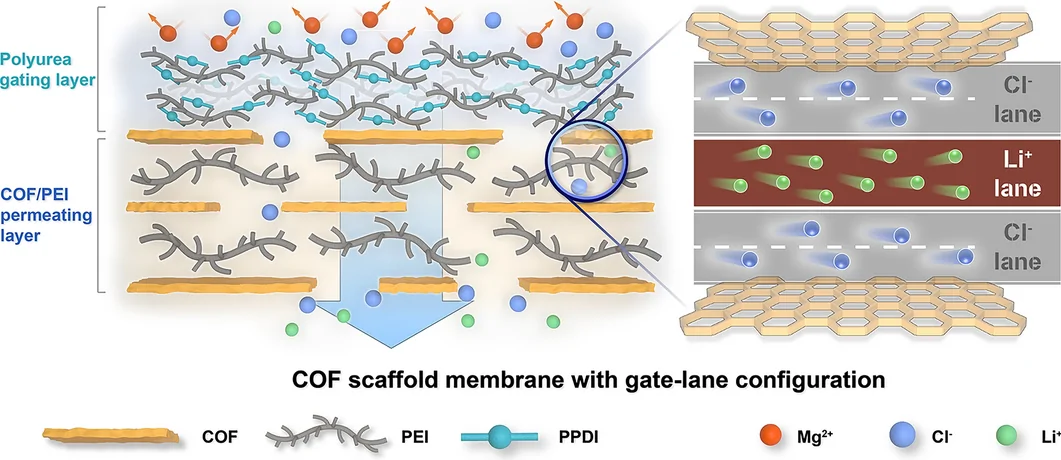

## Introduction

Ion separations meet unprecedented opportunities with the burgeoning new energy source and energy storage technology [1–4]. Membrane technology exhibits great potential in extracting metal ions such as Li^+^ from salt lake brine and seawater [5, 6]. The separation of mono-/divalent ions represents a critical aspect of ion separation, with applications including metal extraction, seawater desalination, and the maintenance of physiological functions. Divalent ions, such as Mg^2+^ and Ca^2+^, are prone to forming scale deposits and thus should be removed in advance to protect reverse osmosis membranes. The balance of ions across cellular membranes, such as Na^+^/K^+^ and Mg^2+^/Ca^2+^, is essential for proper physiological functioning [7–9]. Notably, the separations of mono-/divalent ions play an irreplaceable role in a number of physical and chemical processes involving ions. As for battery electrolyte design and electrochemical deposition, non-target ions of different valences can cause significant interference. Furthermore, certain catalytic reactions and drug activities are exclusively dependent on specific valence ions [10].

In general, ion separations are affected by the electric charges that incur the multiple ion–ion and ion–membrane interactions and thus the complicated ion mixing effect, i.e., the measured perm selectivity or “true selectivity” of a binary ion mixture can significantly deviate from the “ideal selectivity” measured from the permeation flux of each pure ion species [11–15]. To achieve favorable ion mixing effect, which means that the true selectivity is much higher than the ideal selectivity, the innovative and well-defined membrane nanostructure becomes an essential pursuit and critical issue.

Currently, the mechanism of ion mixing effect remains elusive. In most cases, the target ions have competitive interactions with co-ions and counter-ions [16, 17]. To acquire favorable ion mixing effect, high permeation flux of target ions and high rejection to co-ions are desired. The size exclusion and membrane–ion interactions are crucial factors for ion separations, since they directly influence whether ions can readily enter the membrane channels and how fast ions pass through the membrane channels [18, 19]. The existing studies mainly focus on increasing the rejection of co-ions, while the effect of counter-ions on the ion separation performance has been rarely considered. The negative rejection phenomenon derived from electro-neutralization indicates that nexus between target ions and counter-ions could afford favorable ion mixing effect, depending on both material and structure of membranes [20–22]. Owing to the long-range regular channels and rich chemical/structural diversity, covalent organic framework (COF) is expected to become a class of disruptive ion separation membrane materials [23–27]. However, the pore size of COF membranes is often larger than the diameter of ions to be separated, posing a challenge on the membrane structure design.

Herein, we design COF scaffold membrane with gate-lane nanostructure for efficient Li^+^/Mg^2+^ separation. The laminated COF nanosheets bearing quaternary ammonium confer highly positive charge density. When COF nanosheets and polyetherimide (PEI) are co-assembled in the ethanol–water solution through vacuum filtration on the support, PEI is intercalated between COF nanosheets, forming the COF/PEI permeating layer. Subsequently, the permeating layer is soaked in heptane containing 1, 4-phenylene diisocyanate (PPDI), and the cross-linking reaction between PEI and PPDI is triggered, forming the polyurea (PU) gating layer with smaller pores therein. By tuning the mass ratio of COF nanosheets to PEI, the pure PU membrane, COF hybrid membrane, and COF scaffold membrane can be fabricated, respectively.

By manipulating the competition between target ions (Li^+^) and co-ions (Mg^2+^), the gating layer can effectively reject Mg^2+^ and allow for the passage of Li^+^ and counter-ions (Cl^−^) into the permeating layer, affording high Li^+^/Mg^2+^ selectivity. The COF nanosheets serve as the scaffold of the membrane, which provides the spatial foundation for separate pathways of cations and anions. During preparation, the COF scaffold is filled with PEI, forming an asymmetric structure with distinct positive charge density gradients. Due to the different interactions of Li^+^ and Cl^−^ with COF nanosheets and PEI, the individual lanes for Li^+^ and Cl^−^ can be formed within the permeating layer, which is similar to the Na^+^ and Cl^−^ channels of pulmonary epithelial cells [28–34]. As anions are preferentially transported in positively charged channels [35–38], COF nanosheets with higher positive charge density preferentially attract Cl^−^ to form the “Cl^−^ lanes,” leaving sufficient space for Li^+^ transport. Given that cations are suppressed by electrostatic repulsion, PEI with lower positive charge density is chosen to form the “Li^+^ lanes” along PEI chains, facilitating Li^+^ transport. The lane nanostructure is supported by molecular dynamics (MD) simulations, which demonstrates that anions are not merely charge compensators but critical contributors that facilitate Li^+^ transport and improve separation efficiency. This asymmetric charge and spatial configuration enables the lane-separated ion transport mechanism, which is key to achieving efficient Li^+^/Mg^2+^ separation. The optimum COF scaffold membrane displays the superior true Li^+^/Mg^2+^ selectivity of 231.9 with Li^+^ enrichment of 120.2% at the Mg^2+^/Li^+^ mass ratio of 50, exceeding the ideal selectivity of 80.5 and outperforming all the existing positively charged nanofiltration membranes.

## Experimental Section

### Materials

1,3,5-Triformylphloroglucinol (Tp, 98%) was purchased from Jilin Yanshen Technology Co. Ltd. 2,5-Dihydroxyterephthalic acid diethyl ester (≥ 97%) was supplied by Shanghai Dibai Biotechnology Co. Ltd. 1,4-Dibromobutane and potassium iodide, polyethyleneimine (PEI, 10,000 Da, 30 wt% aqueous solution), phenylene diisocyanate (PPDI), magnesium chloride (MgCl_2_, 97%), magnesium sulfate (MgSO_4_, 97%), sodium chloride (NaCl, 99.5%), sodium sulfate (Na_2_SO_4_, 98%), lithium chloride (LiCl, 98%), and lithium sulfate (Li_2_SO_4_, 99%) were bought from Tianjin Heowns Bio-chem Technology Co. Ltd. Potassium carbonate, silver chloride, trimethylamine solution (30 ~ 35 wt% in ethanol), dimethyl sulfoxide, and n-heptane (98%) were purchased from Shanghai Aladdin Bio-chem Technology. Hydrazine hydrate (hydrazine 64%) was supplied by Tianjin Jiangtian Chemicals. Acetone, ethanol, acetic acid, ethyl acetate, and N, N-dimethylformamide were of analytical grade and supplied by Tianjin Real and Lead Chemical Co. Ltd. Polyethylene glycol (PEG) (200, 400, 600, 800, and 1000 Da) was bought from Hefei BASF Biotechnology Co., Ltd. (Anhui, China). Poly(ether sulfone) (PES, pure water permeance≈600 L m^−2^ h^−1^ bar^−1^) supports were obtained from Guo Chu Technology Co., Ltd. (Xiamen, China). Deionized (DI) water was produced by a laboratory water purification device. All solvents and chemicals were of reagent grade and utilized without further purification.

### Preparation of COF Membrane, PU Membrane, COF Scaffold Membrane, and COF Hybrid Membrane

#### Synthesis of COF Nanosheets

The COF nanosheets were synthesized following previous report [26]. DQA monomer (0.06 mmol) was dissolved in the solution of 3 mL dimethyl sulfoxide and 0.6 mL 6 M aqueous acetic acid. Then, Tp monomer (8.41 mg, 0.04 mmol) dissolved in 2 mL dimethyl sulfoxide was added into the as-prepared organic solution of hydrazide monomer dropwise. Next, the resulting mixture was stirred thoroughly. To reach high conversion rate of the monomers, the reaction systems to prepare COF nanosheets were kept undisturbed at 60 °C for 3 days. Afterward, the as-prepared nanosheet colloidal solution was dialyzed in dimethyl sulfoxide for 3 days to remove the residual unreacted monomers. The solution of COF nanosheets was freeze-dried to obtain the nanosheets powder, and the concentration was measured to be about 1 mg mL^−1^.

#### Preparation of COF Membrane

The solution of COF nanosheets was diluted to 5 mL with ethanol and then vacuum filtrated onto PES support membranes.

#### Preparation of PU Membrane

5 mL PEI ethanol solution (0.5 g L^−1^) was vacuum filtrated onto PES support membranes. Subsequently, it was soaked in heptane containing PPDI (0.2 g L^−1^) for 60 s at room temperature. Finally, it was washed by heptane and placed in an oven at 60 °C for 10 min.

#### Preparation of COF Scaffold Membrane and COF Hybrid Membrane

The preparation of COF scaffold membrane and COF hybrid membrane is similar to that of PU membrane. Different volumes of COF nanosheets were added to 5 mL PEI ethanol solution (0.5 g L^−1^) to obtain mass ratio of COF nanosheets to PEI from 0.001 to 0.1 or even higher. The COF scaffold membrane refers to the membrane with the mass ratio of 0.1 and is typical of membranes with high mass ratio. The COF hybrid membrane refers to the membrane with the mass ratio of 0.001 and is typical of membranes with low mass ratio.

### Characterizations

Scanning electron microscope (SEM) images were collected by using a JEOL JSM-IT800 field emission. Transmission electron microscope (TEM) images were collected by the JEOL-2100F instrument. AFM images were taken by a multifunctional scanning probe microscope (NTEGRA Spectra). FTIR patterns were obtained using a BRUKER Vertex 70 equipment. XPS was performed on an ESCALAB Xi + instrument with an Al Kα radiation source. Zeta potential values and Z-equivalent sizes were obtained by a Nano ZS instrument with a 4-mW He–Ne laser. The details of MWCO are given in the Supplementary Information.

### Separation Performance Measurements

The separation performance of the membranes was assessed using a cross-flow filtration setup with an effective separation area of 1.54 cm^2^ at room temperature. The system was allowed to stabilize for 0.5 h under 6.5 bar, after which measurements were taken at 6.0 bar. The reported data represent the average values from three independent experiments for each category. Water permeance (*P*, L m^−2^ h^−1^ bar^−1^) of membranes was calculated via Eq. 1:

1 $$P=\frac{V}{A\Delta t\Delta p}$$

where *V* (L) is the permeated volume, and *A* (m^2^), *∆t* (h), and *∆P* (bar) are the available area of the membrane, the time of the permeation, and the driving pressure, respectively.

Aqueous solutions with 1000 ppm inorganic salt (MgCl_2_, MgSO_4_, NaCl, Na_2_SO_4_, LiCl, and Li_2_SO_4_) were used as feed solutions for selectivity measurements, and the rejection (*R*, %) was calculated via Eq. 2:

2 $$R=\left(1-\frac{{C}_{p}}{{C}_{f}}\right)\times 100\%$$

where ${C}_{p} (\mathrm{ppm})$ and ${C}_{f} (\mathrm{ppm})$ represent the salt concentrations of the permeate and feed solutions, respectively. Salt concentrations were measured using an electrical conductivity meter (Leichi, DDS-11A, China) at 26 ℃, which is linearly related to the conductivity.

Specially, if R<0, it means that ${C}_{p}> {C}_{f}$ and ions are enriched. The enrichment was calculated via Eq. 3:

3 $$E=\left(\frac{{C}_{p}}{{C}_{f}}\right)\times 100\%$$

The selectivity of Li^+^/Mg^2+^ (${S}_{Li,Mg}$), which is also called as separation factor, includes the ideal selectivity in the single-solute system and the true selectivity in the mixed-solute system. The ideal selectivity was calculated via Eq. 4:

4 $${S}_{Li,Mg}=\frac{1-{R}_{Li}}{1-{R}_{Mg}}$$

where ${R}_{Li}$ and ${R}_{Mg}$ are the rejection to LiCl and MgCl_2_, respectively. The concentration of single solution corresponds to that in mixed solution at different Mg^2+^/Li^+^ mass ratios.

The true selectivity was calculated via Eq. 5:

5 $${S}_{Li,Mg}=\frac{{C}_{Li,p}/{C}_{Mg,p}}{{C}_{Li,f}/{C}_{Mg,f}}$$

where ${C}_{Li,p}$, ${C}_{Mg,p}$, ${C}_{Li,f},$ and ${C}_{Mg,f}$ are the LiCl concentration in feed solution, the MgCl_2_ concentration in feed solution, the LiCl concentration in permeate solution, and the MgCl_2_ concentration in permeate solution, respectively. The selectivity of the membranes was assessed using the concentrations of corresponding ions in the feed and permeate solutions, measured by ion chromatography (IC, Thermo ICS-1100, USA).

## Results and Discussion

### Preparation and Structures of COF Scaffold Membrane

**Fig. 1:**
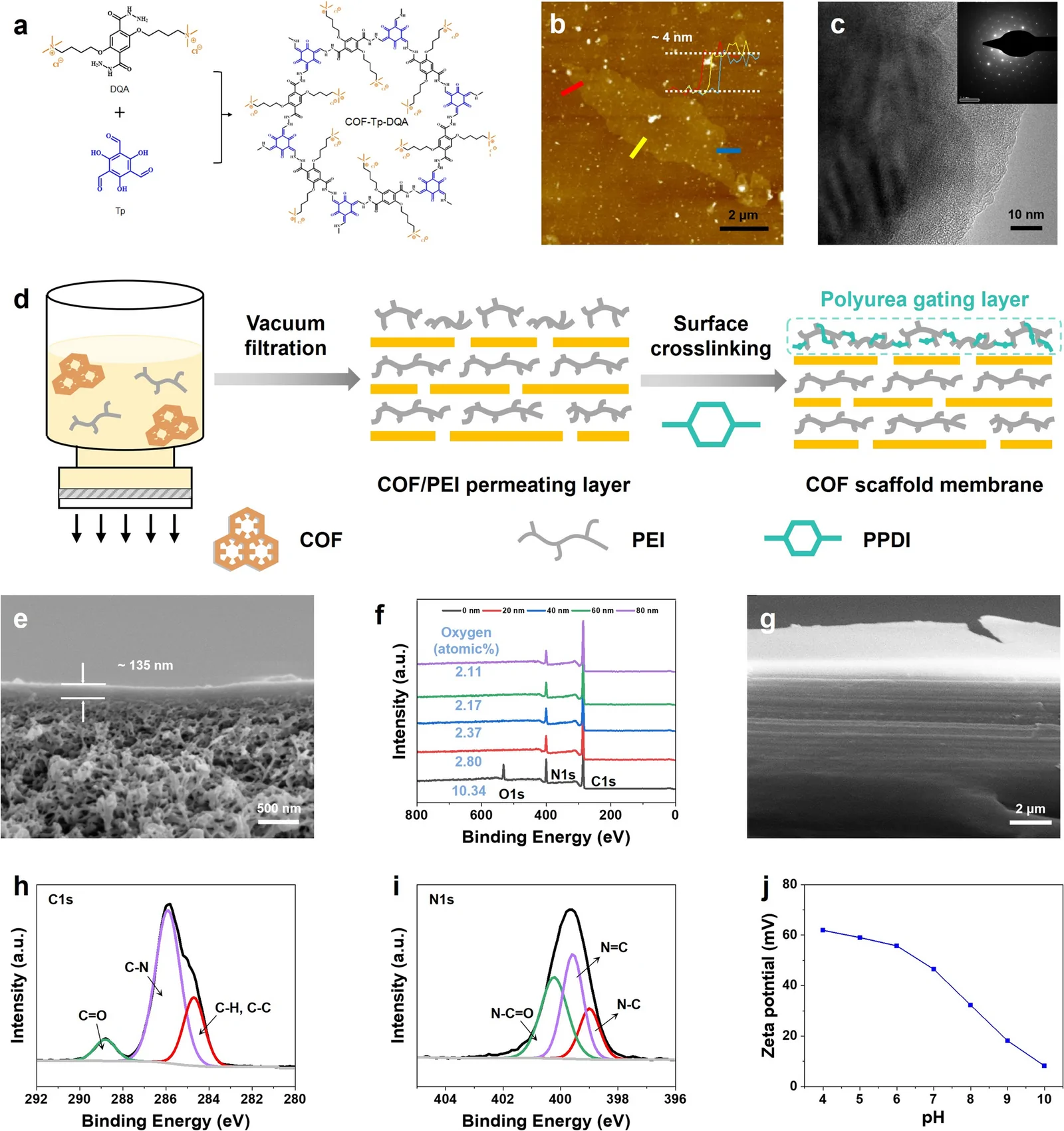
Preparation and structures of COF scaffold membrane. **a** Schematic illustration of the synthesis process of COF. **b** AFM image and **c** TEM image of COF nanosheets. **d** A scheme illustrating the preparation of COF scaffold membrane. **e** Cross-sectional SEM images of COF scaffold membranes and **f** XPS depth profile of COF scaffold membrane. **g** Cross-sectional SEM images of thicker COF scaffold membrane at the same COF/PEI mass ratio. XPS survey spectra showing **h** C 1*s* spectra and **i** N 1*s* spectra of COF scaffold membrane. **j** Surface zeta potential of COF scaffold membrane

The COF nanosheets composed of 4,4'-((2,5-di(hydrazinecarbonyl)-1,4-phenylene)bis(oxy))bis(N,N,N-trimethylbutan-1-aminium (DQA) and 1,3,5-triformylphloroglucinol (Tp) were prepared following Fig. 1a. Nanosheets with a lateral dimension of about 4–6 µm and thickness of about 4 nm are obtained, as shown in the atomic force microscopy (AFM) and transmission electron microscopy (TEM) images (Figs. 1b and S1). From high-resolution transmission electron microscopy (HRTEM) images, continuously distributed lattice fringes are observed, proving the formation of crystal structures (Fig. 1c). The simulation via Material Studio shows that the pore size of COF is ~ 1.6 nm (Fig. S2). The COF nanosheets are positively charged with a zeta potential of around + 35 mV.

The as-synthesized COF nanosheets were dispersed in a mixture of water and ethanol and then mixed with PEI before filtration through the ultrafiltration polyethersulfone (PES) support, forming the COF/PEI permeating layer. Then, the COF/PEI layer was soaked in the heptane solution containing PPDI, triggering the cross-linking reaction between PEI and PPDI on the surface and forming the PU gating layer (Fig. 1d). The mass ratio of COF nanosheets to PEI was varied between 0 and 1. Specifically, the membranes with the mass ratios of 0.001 and 0.1 are labeled as COF hybrid membrane and COF scaffold membrane, respectively. COF membranes are prepared from pure COF nanosheets.

Compared with pure COF, pure PU, and COF hybrid membrane, the surface of COF scaffold membrane is the smoothest, with a low roughness (Ra) of 0.610 nm (Figs. S3–S10), implying that the intercalation of PEI into COF nanosheets directly affects the formation of PU layer. The thickness of pure COF membrane is ~ 80 nm. After intercalating PEI and forming the PU gating layer, the thickness of COF scaffold membrane becomes ~ 135 nm (Figs. 1e and S11–S13). The thickness of gating layer was measured by X-ray photoelectron spectroscopy (XPS) depth profile (Fig. 1f). At the surface, the ratio of oxygen atoms is 10.34%. After etching the membrane, this ratio drops to 2.80% at the depth of 20 nm and remains at ~ 2% at larger depths, which is consistent with theoretical ratio of the COF/PEI permeating layer before cross-linking with PPDI. It indicates that the thickness of PU gating layer is less than 20 nm. To observe the cross-sectional structure of the COF scaffold membrane, we prepared a thicker membrane at the same COF/PEI mass ratio. Cross-sectional SEM images show that the COF scaffold membrane exhibits well-defined laminated microstructure similar to the brick-and-mortar structure of nacre, verifying the role of COF nanosheets as the scaffold (Fig. 1g) [39, 40]. For the PEI intercalated between the COF nanosheets in the bulk scaffold (i.e., not on the surface exposed to PPDI), the dominant interaction is hydrogen bonding. The abundant amine groups (−NH₂/−NH-) of PEI can form multiple hydrogen bonds with C = O and C–N of the COF nanosheets [41].

The chemical structure of the gating layer (Scheme S2) was explored by attenuated total reflection-Fourier transform infrared spectroscopy (ATR-FTIR, Fig. S14) and XPS (Fig. 1h, i). The primary amine groups (−NH_2_) of PEI on the membrane surface react with the isocyanate groups (−N = C = O) of PPDI to form urea linkages (−NH–CO–NH-), creating the polyurea cross-linked gating layer. The absorption peak observed at 1656 cm^−1^ is attributed to the stretching vibration of the carbonyl groups in urea groups. There are typically two characteristic absorption signals: 1735 cm^−1^ for the free carbonyl group and 1656 cm^−1^ for the carbonyl group bonded with -NH- through hydrogen bonding. The absence of peaks at 1735 cm^−1^ implies the abundance of hydrogen bonds in the PU layer, enabling a relatively dense structure. Furthermore, the C1s core-level spectra can be deconvoluted into three different peaks with binding energies of 284.7, 285.9, and 287.8 eV, which are ascribed to C–H/C–C, C–O, and O = C–O/O = C–N, respectively (Fig. 1h). In Fig. 1i, the N 1*s* can be fitted to peaks at 399.8, 400.5, and 401.6 eV assigning to N–C, N = C, and N–C = O, respectively, further confirming the formation of PU gating layer [42]. The ratio of oxygen to nitrogen can represent the cross-linking degree of the polyurea layer. The XPS elemental analysis shows an O/N atomic ratio of 0.65 of the polyurea layer, suggesting a high degree of polyurea formation between PPDI and PEI.

The molecular weight cutoff (MWCO) of the COF scaffold membrane is 335 Da (Fig. S15), and the corresponding pore radius is 0.42 nm, which is between the hydrated radius of Li^+^ (0.340 nm) and that of Mg^2+^ (0.428 nm) [43]. The surface zeta potential remains always positive even when the pH increases to 10 (Fig. 1j). At neutral pH, as the mass ratio of COF nanosheets to PEI grows from 0 to 0.1, the zeta potential increases from 50.2 to 55.7 mV (Fig. S16). Less PEI participates in the reaction to form PU at higher mass ratio imply that residual positive charges from PEI chains decrease, and the higher zeta potential is arisen from the COF nanosheets (Fig. S17).

### Separation Performance of COF Scaffold Membrane

**Fig. 2:**
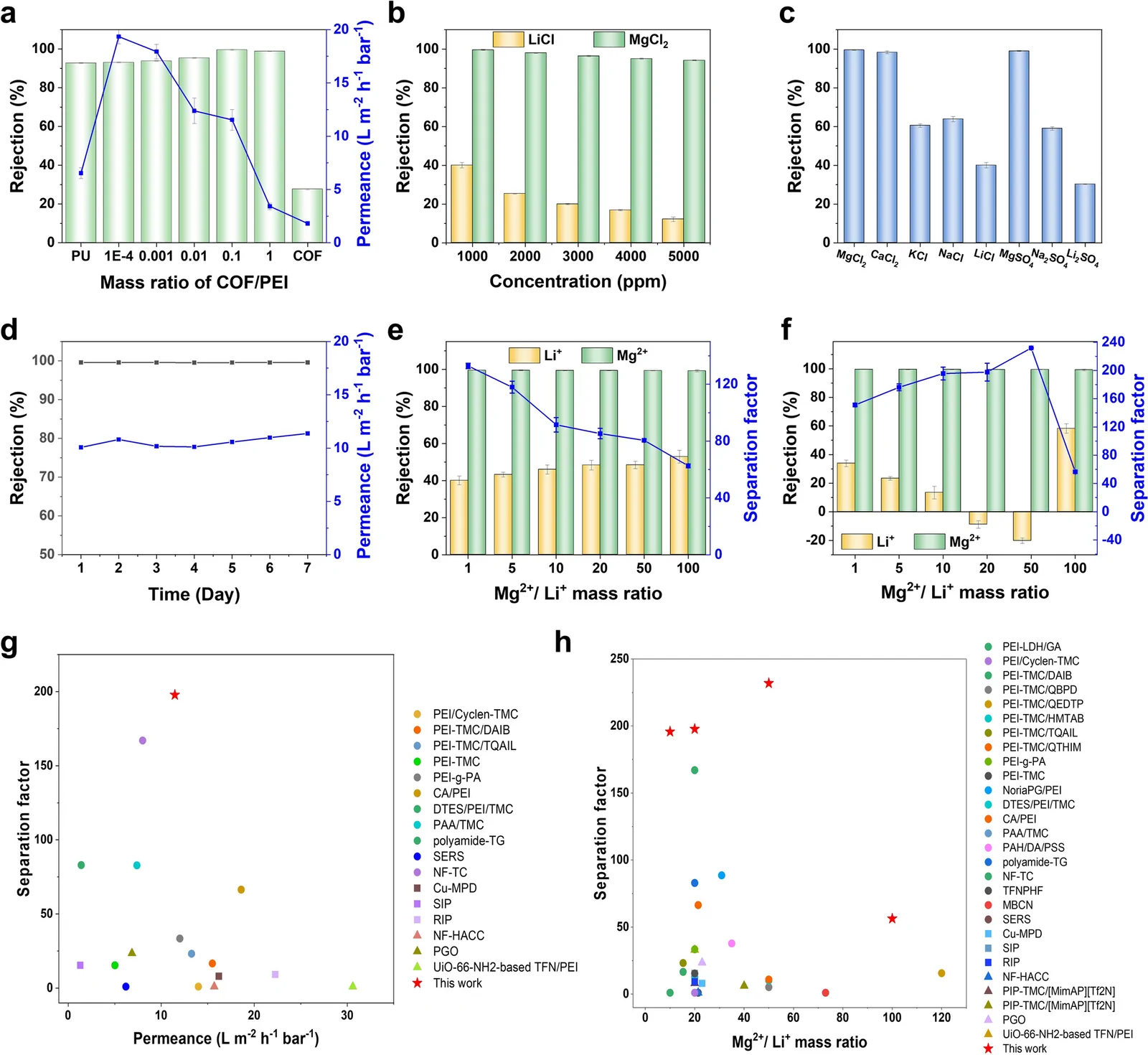
Separation performance of COF scaffold membrane. **a** Effect of mass ratio of COF/PEI on performance of COF scaffold membrane. **b** Rejections to MgCl_2_ and LiCl with respect to different feed solution concentrations. **c** Rejections to different salt solutions. **d** Operational stability for a long term. The feed solution is 1000 ppm MgCl_2_. Rejection and separation factor in **e** single-solute and **f** mixed-solute system. The total concentration of mixed feed solution is 2000 ppm. The concentration of single solution corresponds to that in mixed solution. **g** Separation factor and permeance of COF scaffold membrane and the reported nanofiltration membrane. The Mg^2+^/Li^+^ mass ratio of mixed feed solution is 20. **h** Comparison of separation factor of the reported nanofiltration membranes with different Mg^2+^/Li^+^ mass ratio

To evaluate the separation performance in a single-solute system, water permeance and salt rejection were measured using a laboratory-scale cross-flow filtration setup. With rising mass ratio of COF nanosheets to PEI till 0.1, the MgCl_2_ rejection increases from 92.7% to 99.5% (Fig. 2a). This is due to the higher zeta potential of the membranes that intensifies the Donnan effect to reject co-ions. However, excess COF nanosheets will introduce larger mass transport resistance in the permeating layer. With the ratio increasing from 0.1 to 1, the permeance decreases from 11.5 to 3.4 L m^−2^ h^−1^ bar^−1^. Additionally, the structure of COF scaffold membrane was optimized by adjusting the concentration of PPDI, volume ratio of water to ethanol, and reaction time (Figs. S18–S20). The optimum membrane performance is achieved at 0.2 g L^−1^ PPDI, 100% ethanol, and reacting 1 min. To further understand the ion transport mechanisms through the membrane, rejections to different salt solutions were measured. The rejection follows the sequence of MgCl_2_ (99.5%)≈MgSO_4_ (99.0%) > CaCl_2_ (98.4%) > NaCl (63.9%) > KCl (60.6%) > Na_2_SO_4_ (59.0%) > LiCl (40.1%) > Li_2_SO_4_ (30.2%) (Fig. 2c), which typically shows much lower rejection to LiCl and Li_2_SO_4_ than MgCl_2_ and MgSO_4_, highlighting an efficient Li^+^/Mg^2+^ separation capacity. This is attributed to the synergistic effect of size exclusion and electrostatic repulsion on ion transport through the COF scaffold membrane with appropriate pore size and high positive charges.

The COF scaffold membrane also demonstrates excellent stability under high salt concentration, high pressure, and long-term operation. Rejections to 1000 ppm MgCl_2_ and LiCl are 99.5% and 40.1% (Figs. 2b and S21–S22), respectively. When the MgCl_2_ concentration increases from 1000 to 5000 ppm, the rejection slightly declines from 99.5 to 94.2%, respectively. The declining trend can be attributed to the charge screening effect from the accumulation of anions. Considering that realistic salt lake brines often contain Mg^2+^ above 5000 ppm, the high rejection rates exceeding 94% make COF scaffold membrane highly promising for practical applications. As shown in Fig. S23, the rejection to MgCl_2_ remains at 99% against different pressures, and the pure water flux is almost linearly related to the pressure. Figure 2d displays stable separation performances over the test duration of 7 days. Additionally, the membrane exhibited outstanding stability in H_2_SO_4_ (pH = 3) and NaOH (pH = 11) conditions for 7 days (Fig. S24).

Furthermore, to evaluate the ion mixing effect of the COF scaffold membrane, separation experiments were carried out using 2000 ppm MgCl_2_-LiCl mixed solutions with different Mg^2+^/Li^+^ mass ratios as the feeding solution. The separation performances in single-solute system were also evaluated under the corresponding concentrations for comparison. In the single-solute system (Fig. 2e), with the Mg^2+^/Li^+^ ratio varying from 1 to 100, the Mg^2+^ rejection decreases from 99.5% to 99.2% and the Li^+^ rejection increases from 40.1% to 52.9%, conferring the declined ideal separation factor from 132.9 to 62.6. In contrast, when the mixed-solute solutions were tested (Fig. 2f), the rejection to MgCl_2_ remains stable, while the rejection to LiCl changes considerably with the increase of the Mg^2+^/Li^+^ ratio. As the Mg^2+^/Li^+^ ratio grows from 1 to 50, the Li^+^ rejection decreases from 33.9% to −20.2% and the true separation factor increases from 151.2 to the highest value of 231.9, exceeding the idea selectivity remarkably. The remarkable enhancement in true selectivity, significantly surpassing the ideal value, originates from a favorable ion mixing effect. In the mixed solution, Li^+^, with its smaller hydrated radius, more effectively competes for and occupies the transport pathways in the gating layer. This competitive occupation not only facilitates Li^+^ permeation but also further sterically and electrostatically hinders the access of the larger Mg^2+^, thereby amplifying its rejection beyond the level observed in the single-solute system. Across a wide range of feed ratios (from 1 to 50), the Mg^2+^/Li^+^ mass ratio in the permeate is drastically reduced to values below 0.22, which is over 200 times lower than the corresponding feed ratio (Table S1). Further increase of the Mg^2+^/Li^+^ ratio to 100 leads to a higher Li^+^ rejection of 58.2% and hence lowers true separation factor of 56.3. Remarkably, the true selectivity is higher than the ideal selectivity in the range of Mg^2+^/Li^+^ ratio from 1 to 50, which indicates the COF scaffold membrane generates the favorable ion mixing effect, which will be further explored via molecular dynamic (MD) simulation in the next section.

In the simulated salt like brines (Fig. S25), the rejection for divalent ions (Mg^2+^, Ca^2+^) remains exceptionally high and stable compared to their single-solute values (99.5% and 98.4%, respectively). For Li^+^, the rejection is predicted to drop sharply from 40.1% to 5.2%, which is the signature of the favorable ion mixing effect. These results strongly underscore the great application potential of the COF scaffold membrane for lithium extraction from real salt lake brines. We compared the true separation factor of the COF scaffold membrane with those reported in other literature (Table S2). As shown in Fig. 2g, h, our membrane shows superior separation factor under different Mg^2+^/Li^+^ mass ratios.

### Ion Mixing Effect Analysis in COF Scaffold Membrane

**Fig. 3:**
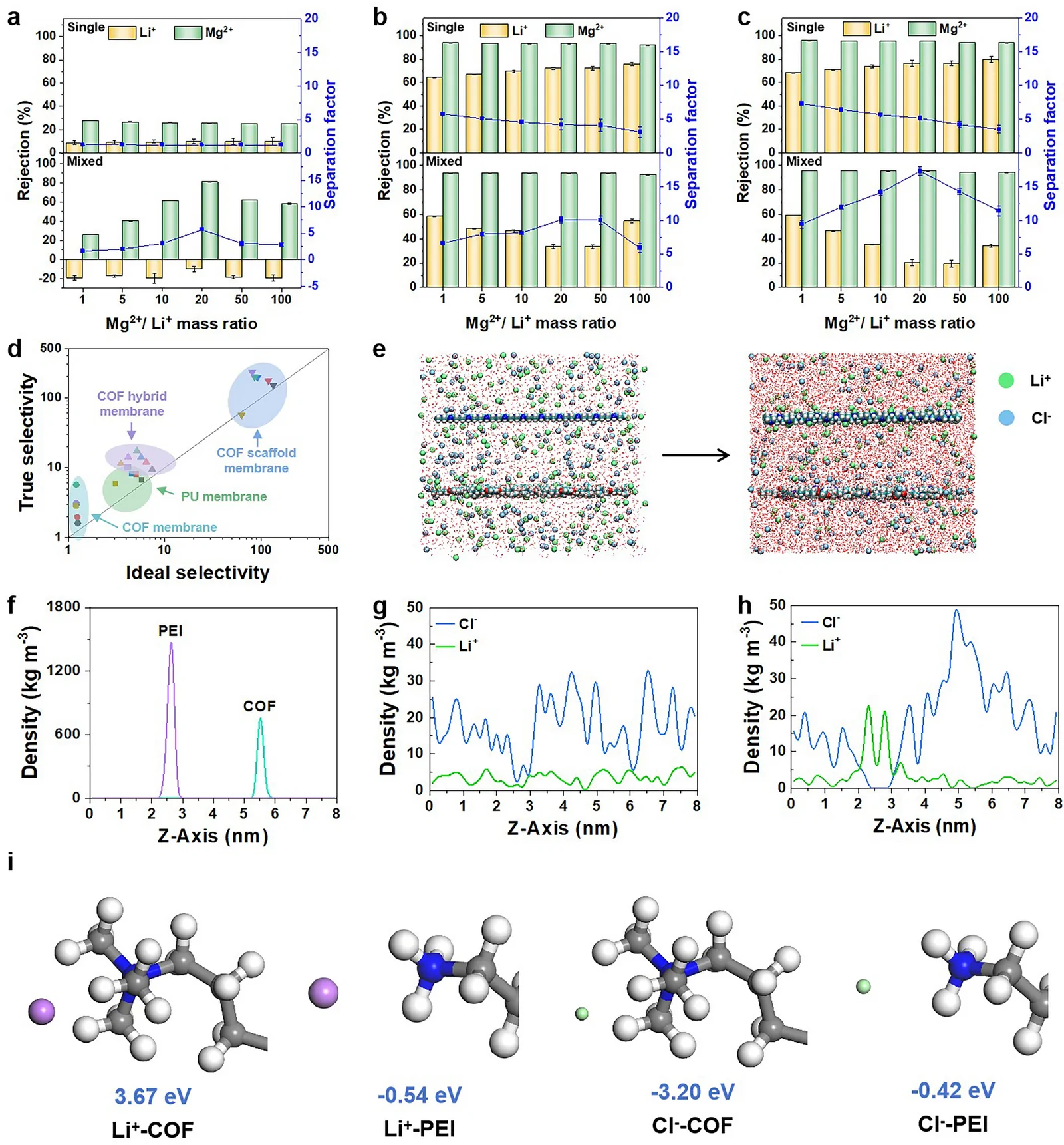
Separation performance of comparison sample membranes and MD simulation of ion distribution asymmetry in COF scaffold membrane. Rejection and separation factor of **a** COF membrane, **b** PU membrane, and **c** COF hybrid membrane. **d** True selectivity and ideal selectivity of four membranes. **e** Initial MD model and model at equilibrium. **f** Density of COF nanosheets and PEI in the Z direction. Density of Cl^−^ and Li^+^ in the Z direction at **g** 0 ns and **h** 50 ns. The feed solutions are MgCl_2_ and LiCl. **i** Interaction energy calculated by DFT simulation between Li^+^/Cl^−^ and COF/PEI

To explore the effect of the membrane nanostructure on the ion mixing effect and separation mechanism, the separation performances of COF membrane (Fig. 3a), PU membrane (Fig. 3b), and COF hybrid membrane (Fig. 3c) in single-solute and mixed solution system were evaluated. PU membrane, COF hybrid membrane, and COF scaffold membrane exhibit similar trend but different degree of favorable ion mixing effect. Notably, the COF membrane exhibits low separation factors, attributed to the larger pore size with poor Mg^2+^ sieving capacity. However, in mixed-solute system, the COF membrane exhibits enhanced rejection to Mg^2+^ and negative rejection to Li^+^, suggesting that the highly positively charged COF nanosheets can drag large amount of Cl^−^ across the membrane and lead to the accelerated Li^+^ permeation. PU and COF hybrid membranes also exhibit higher true selectivity than ideal selectivity, but their favorable ion mixing effect is not so pronounced as that of the COF scaffold membrane (Fig. 3d).

The significantly favorable ion mixing effect generated by COF scaffold membrane can be ascribed to the competitive interactions among target ions (Li^+^), co-ions (Mg^2+^), and counter-ions (Cl^−^) by the gate-lane nanostructure. The “gate” can utilize the competition between target ions and co-ions to acquire favorable ion mixing effect. For the COF scaffold membrane with appropriate pore size and highly positive charges, in the mixed-solute system, Li^+^ with lower co-ion charge and smaller hydrated radius is easier to enter and take up the membrane channel, preventing Mg^2+^ from entering the membrane channel and thus acquiring favorable ion mixing effect. For PU and COF hybrid membranes, the lower rejections to Mg^2+^ than COF scaffold membrane render lower true selectivity and less favorable ion mixing effect.

It is worth noting that the Li^+^ rejection of COF scaffold membrane is lower than that of PU membrane and COF hybrid membrane and even becomes negative value at high Mg^2+^/Li^+^ mass ratio. The permeating layer with “lanes” nanostructure greatly contributes to the high permeation flux of Li^+^ in COF scaffold membrane. Regulating either anions or cations can influence the co-transport of anions and cations, resulting in different permeability of salt [44]. For channels of positively charged membranes, the transport of anions is the rate-determining step [45]. To leverage the interactions between target ion and counter-ion, we design the “lanes” for counter-ions to transport synergistically with target ions. Due to ultrahigh rejection to Mg^2+^, the vast majority ions in the permeating layer are Li^+^ and Cl^−^. The lanes in the permeating layer comprise the individual lanes for target ions (Li^+^) and counter-ions (Cl^−^) on the basis of charge asymmetry effect, which can mediate the distance and interactions between ions and the membrane [28–33]. As anions are more preferentially transported in positively charged channels [16, 35–38], the COF nanosheets with higher positive charge density preferentially attract Cl^−^ to form the “Cl^−^ lanes,” leaving sufficient space for low-resistance Li^+^ transport. Given that cations are suppressed by electrostatic repulsion, PEI with lower positive charge density is chosen to form the “Li^+^ lanes” along PEI chains, facilitating Li^+^ transport.

MD simulations were carried out to prove the asymmetric ion distribution within the permeating layer of the COF scaffold membrane. In the initial state, Cl^−^ and Li^+^ are randomly and uniformly distributed throughout the box containing the COF nanosheets and PEI. After 50 ns of simulation, it is observed that Cl^−^ tends to accumulate more toward the COF nanosheets layer, while the Li^+^ is adsorbed more around the PEI (Figs. 3e and S26). We employ the radial distribution function (RDF) to further investigate the interactions of Li^+^ and Cl^−^ in the system with the COF and PEI as well as the corresponding preferential aggregation behavior. RDFs between each of the two types of ions and the COF nanosheets and PEI are individually calculated. Figure S27 illustrates the RDF distribution of Cl^−^. There is a distinct RDF peak between Cl^−^ and COF nanosheets, whereas no notable RDF peak is observed between Cl^−^ and PEI. This suggests that Cl^−^ has a stronger tendency to aggregate around the COF nanosheets. Figure S28 shows the RDF distribution of Li^+^. In contrast to Cl^−^, a clear RDF peak is present between Li^+^ and PEI, while no significant RDF peak exists between Li^+^ and COF nanosheets, indicating that Li^+^ preferentially aggregates around the PEI. To further verify the difference in the distribution of the two types of ions around the COF nanosheets and PEI, the density profiles in the Z direction are analyzed for the COF nanosheets and PEI as well as the two ions before and after the simulation (Fig. 3f–h). After 50 ns of simulation, Li^+^ exhibits a pronounced enrichment density peak near the PEI, indicating that the majority of Li^+^ accumulate around the PEI. Conversely, Cl^−^ exhibits a distinct enrichment density peak near the COF nanosheets, suggesting that Cl^−^ primarily aggregates around the COF nanosheets. In summary, these findings demonstrate that in this mixed system, Cl^−^ preferentially enriches around the COF nanosheets, while Li^+^ tends to accumulate near the PEI. Notably, the diffusion coefficients of these two types of ions within the system are shown in Fig. S29 and compared with other works in Table S3. The diffusion coefficient of Cl^−^ is D = 2.1405 × 10^–5^ cm^2^ s^−1^, and the diffusion coefficient of Li^+^ is D = 0.9888 × 10^–5^ cm^2^ s^−1^. The high diffusion coefficients of these two ions not only lead to the asymmetric distribution and space occupancy, but also facilitate ion transport in their own lanes [46–50]. These findings demonstrate that anions are not merely charge compensators but critical contributors that facilitate Li^+^ transport and improve separation efficiency.

Meanwhile, DFT simulations were carried out to calculate the interaction energies. Firstly, the Li^+^**–**COF interaction is strongly repulsive (3.67 eV) due to electrostatic repulsion, preventing Li^+^ from approaching the COF. This repulsive force also effectively pushes Li^+^ toward regions with lower positive charge density, achieving the spatial separation of Li^+^ and Cl^−^. Secondly, the Li^+^**–**PEI interaction is weakly attractive (−0.54 eV), which represents a moderate weak ion**–**dipole or coordination interaction. For unprotonated amines, the electronegative nitrogen atom carries a partial negative charge, creating a dipole moment that allows weak ion–dipole interactions with Li^+^. Under specific conditions, lone pair electrons may even form weak coordination with Li^+^. When protonated, the amines electrostatically repel Li^+^. Overall, the repulsion from the partially protonated amine groups on PEI is much weaker than that from the quaternary ammonium groups on COF. This relatively friendly environment provides a low-resistance transport channel—the Li^+^ lanes—allowing Li^+^ to diffuse rapidly along PEI chains facilitated by weak interactions with the amines and their hydration layers. Thirdly, the Cl^−^-COF interaction is strongly attractive (−3.20 eV) due to electrostatic attraction, which is sufficient to partially disrupt the hydration shell of Cl^−^, enabling it to approach the quaternary ammonium groups closely. This is the core driving force for forming the Cl^−^ lanes and thus ensures the preferential and rapid transport of Cl^−^ through the COF domains, maintaining electroneutrality and cooperatively promoting Li^+^ transport by avoiding excessive counter-electromotive force. Finally, the Cl^−^-PEI interaction is weakly attractive (−0.42 eV), involving moderate electrostatic attraction and hydrogen bonding, but it is much weaker than that near COF nanosheets due to the lower charge density. This further reinforces the tendency for Cl^−^ to be transported within the Cl^−^ lanes of the COF rather than in the Li^+^ lanes of the PEI, maintaining the spatial asymmetry of ion transport. In summary, within the COF scaffold membrane, the combination of strong attraction between quaternary ammonium groups and Cl^−^ and strong repulsion between quaternary ammonium groups and Li^+^ achieves the initial spatial separation of ions. The weak interaction between amine groups and Li^+^ provides a dedicated fast transport channel for Li^+^. This synergistic effect, based on charge asymmetry, is the fundamental reason why the gate-lane nanostructure achieves extremely high selectivity and flux. The density profiles (Fig. 3g, h) from the MD simulations, which show Cl^−^ biased toward COF and Li^+^ biased toward PEI, visually validate the final outcome of these interactions.

Compared with the COF scaffold membrane, the PU membrane has randomly cross-linked bulk structure. For COF hybrid membrane, the content of COF nanosheets is too low to form continuous lanes within the polyurea matrix. Consequently, the random transport of Li^+^ and Cl^−^ can lead to lower transport efficiency within the PU and COF hybrid membranes, resulting in higher Li^+^ rejections and lower true selectivity. In addition, the separation performances of COF scaffold membrane, PU membrane, COF hybrid membrane, and COF membrane in the solution of MgSO_4_ and Li_2_SO_4_ were also evaluated (Figs. S30–S37), which is consistent with that in the solution of MgCl_2_ and LiCl. The COF scaffold membrane exhibits the true selectivity of 123.8 at the Mg^2+^/Li^+^ mass ratio of 5.

## Conclusions

In summary, we design a kind of COF scaffold membrane with gate-lane nanostructure, which exhibit favorable ion mixing effect and achieve superior Li^+^/Mg^2+^ selectivity. The gating layer in COF scaffold membrane, bearing smaller pore size, affords high rejection to co-ions (Mg^2+^) and thus high Li^+^/Mg^2+^ selectivity. The permeating layer in COF scaffold membrane, asymmetric charge, and spatial nanostructure bearing large pore size and individual lanes for Li^+^ and Cl^−^, facilitates Li^+^ transport and thus high Li^+^ permeability. The optimum COF scaffold membrane exhibits the true Li^+^/Mg^2+^ selectivity of 231.9 with Li^+^ enrichment of 120.2% at the Mg^2+^/Li^+^ mass ratio of 50, exceeding the ideal selectivity of 80.5 and outperforming all the existing positively charged nanofiltration membranes. This work will inspire the rational design of membrane structure to achieve favorable mixing effect and break the membrane permeability**–**selectivity trade-off in ion separation applications.

## Supplementary Information

Below is the link to the electronic supplementary material.

Supplementary file1 (DOCX 10731 kb)
