# Supplementary material for: COF Scaffold Membrane with Gate-Lane Nanostructure for Efficient Li+/Mg2+ Separation
Source: Nanomicro Lett. 2026 Jan 2;18:126. doi: 10.1007/s40820-025-01972-1 (PMC12757503; doi:10.1007/s40820-025-01972-1)
Supplement: Supplementary file 1 — Supplementary file1 (DOCX 10731 kb) [file 40820_2025_1972_MOESM1_ESM.docx]

Supporting Information for

**COF Scaffold Membrane with Gate-Lane Nanostructure for Efficient Li^+^/Mg^2+^ Separation**

Zixuan Zhang^1,2^, Yan Kong^3^, Runlai Li^5^, Xiaolin Yue^3^, Hao Deng^1,2^, Yu Zheng^3,4^, Sui Zhang^2*^, Runnan Zhang^3,4*^, Zhongyi Jiang^1,3,4,6*^

^1^Joint School of National University of Singapore and Tianjin University, International Campus of Tianjin University, Binhai New City, Fuzhou 350207, P. R. China

^2^Department of Chemical and Biomolecular Engineering, National University of Singapore, 4 Engineering Drive 4, Singapore 117585, Singapore

^3^Key Laboratory for Green Chemical Technology of Ministry of Education, School of Chemical Engineering and Technology, Tianjin University, Tianjin 300072, P. R. China

^4^Ningbo Key Laboratory of Green Petrochemical Carbon Emission Reduction Technology and Equipment, Zhejiang Institute of Tianjin University, Ningbo, Zhejiang 315201, P. R. China

^5^College of Polymer Science & Engineering, State Key Laboratory of Polymer Materials Engineering, Sichuan University, Chengdu 610065, P. R. China

^6^State Key Laboratory of Synthetic Biology, Tianjin University, Tianjin 300072, P. R. China

*Corresponding authors. E-mail: [zhyjiang@tju.edu.cn](mailto:zhyjiang@tju.edu.cn) (Zhongyi Jiang), [runnan.zhang@tju.edu.cn](mailto:runnan.zhang@tju.edu.cn) (Runnan Zhang), [chezhangsui@nus.edu.sg](mailto:chezhangsui@nus.edu.sg) (Sui Zhang)

**S1 Supplementary Experimental Methods**

**S1.1 Synthesis of 4,4'-((2,5-di(hydrazinecarbonyl)-1,4-phenylene)bis(oxy))bis(N,N,N-trimethylbutan-1-aminium) (DQA) monomer**

DQA monomer was synthesized according to our previous study [S1].

*diethyl 2,5-bis(4-bromobutoxy)terephthalate* (A). 2,5-dihydroxyterephthalic acid diethyl ester (3.05 g, 12.0 mmol), potassium carbonate (8.29 g, 0.06 mol) and potassium iodide (450 mg, 2.7 mmol) were dissolved in 250 mL of acetone, to which 1,4-dibromobutane (10.36 g, 48.0 mmol) was added. Afterwards, the resulting mixture was heated at 70 °C for 27 h under N_2_ protection and then cooled to room temperature. After filtration and filtrate evaporation, the crude product was purified by the chromatographic column to obtain A as a white solid (3.63 g, 6.93 mmol, 57.8% yield). ^1^H NMR (500 MHz, CDCl_3_): δ = 7.34 (s, 1H), 4.37 (q, *J* = 7.1 Hz, 2H), 4.05 (t, *J* = 6.0 Hz, 2H), 3.50 (t, *J* = 6.6 Hz, 2H), 2.15 – 2.06 (m, 1H), 1.97 (p, *J* = 6.9, 6.3 Hz, 1H), 1.39 (t, *J* = 7.1 Hz, 3H). ^13^C NMR (101 MHz, CDCl_3_): δ = 165.88, 151.76, 124.99, 116.81, 68.90, 61.48, 33.49, 29.53, 28.01, 14.46.

*4,4'-((2,5-bis(ethoxycarbonyl)-1,4-phenylene)bis(oxy))bis(N,N,N-trimethylbutan-1-aminium)* (B). A (3.63g, 6.93 mmol) was dissolved in 50 mL of ethanol and 15 mL of trimethylamine solution was added. The resulting mixture was refluxed at 50 °C for 36 h. Through solution evaporation, the obtained solid was dissolved in 40 mL of water, to which silver chloride (2.15 g, 15 mmol) was added. The mixture was stirred at 90 °C for 7 h under N_2_ protection. After cooling, the mixture was filtered and the filtrate was evaporated to obtain B as a white solid (3.76 g, 6.8 mmol, 98.1% yield). ^1^H NMR (500 MHz, DMSO-*d*_6_): δ = 7.34 (s, 1H), 4.29 (q, J = 7.1 Hz, 2H), 4.05 (t, J = 6.0 Hz, 2H), 3.44 – 3.38 (m, 2H), 3.07 (s, 9H), 1.86 (dt, J = 15.7, 7.6 Hz, 2H), 1.73 (p, J = 6.6 Hz, 2H), 1.30 (t, J = 7.1 Hz, 3H). ^13^C NMR (101 MHz, DMSO-*d*_6_): δ = 165.10, 150.51, 124.70, 115.72, 68.48, 64.76, 60.97, 52.05, 25.60, 18.47, 14.10.

*4,4'-((2,5-di(hydrazinecarbonyl)-1,4-phenylene)bis(oxy))bis(N,N,N-trimethylbutan-1-aminium)* (DQA). B (3.76 g, 6.8 mmol) was dissolved in 60 mL of ethanol and 10 mL of hydrazine hydrate was added. The solution was heated to reflux at 80 °C for 48 h. After cooling, the mixture was evaporated and the product was then vacuum-dried at 45 °C for 12 h to obtain DQA as a faint yellow solid (3.43 g, 6.53 mmol, 96.0% yield). ^1^H NMR (500 MHz, DMSO- *d*_6_): δ = 9.33 (s, 1H), 7.27 (s, 1H), 4.56 (s, 2H), 4.08 (t, J = 5.9 Hz, 2H), 3.47 – 3.36 (m, 2H), 3.08 (s, 9H), 1.86 (dt, J = 15.7, 7.1 Hz, 2H), 1.76 (p, J = 7.2, 6.7 Hz, 2H). ^13^C NMR (101 MHz, D_2_O): δ = 167.16, 149.96, 126.43, 115.04, 69.11, 66.18, 52.93, 25.27, 19.52.


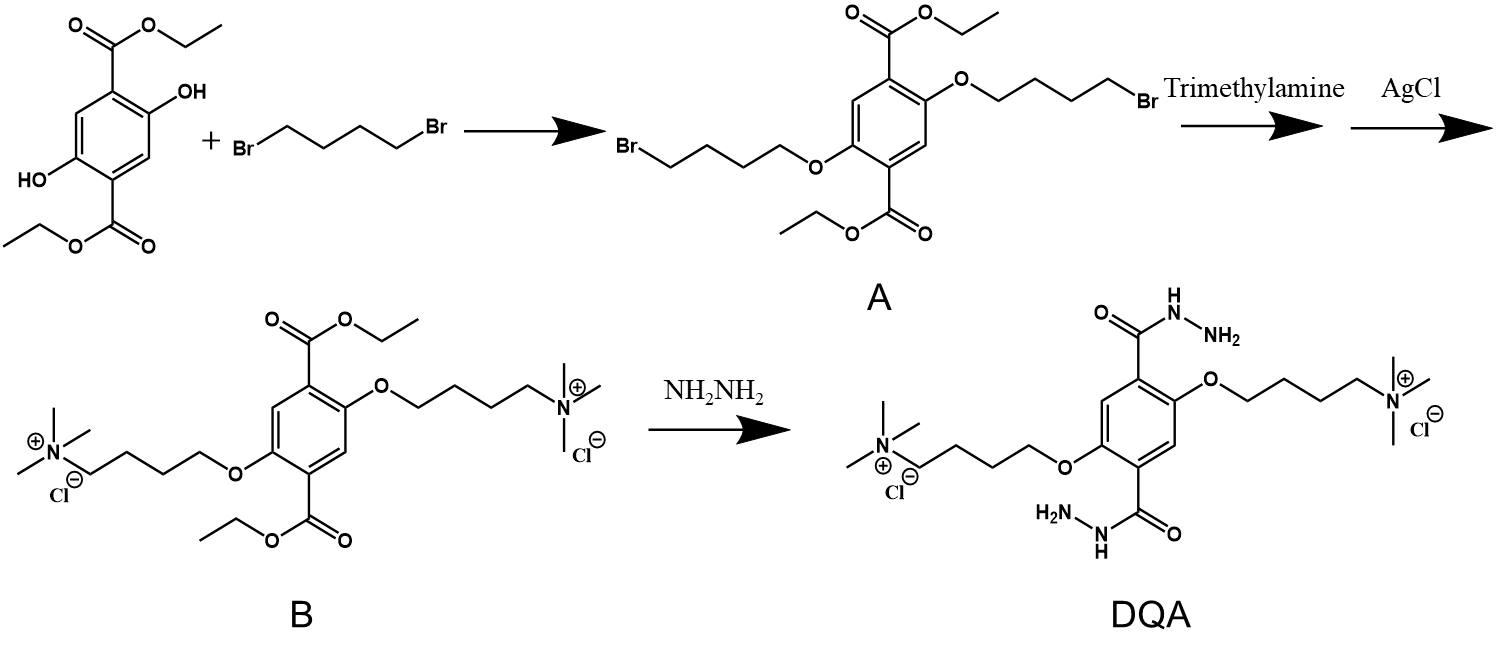


**Scheme S1** Synthesis of DQA monomer

**S1.2 Determination of MWCO of membranes**

The MWCO of membranes was identified by the rejection of 90% for spherical neutral solutes polyethylene glycol (PEG) with a molecular weight of 200, 400, 600, 800, and 1000 Da. The experiments were conducted under the operating conditions of 200 ppm feed solution under 4.0 bar pressure. The rejection of solutes was calculated using Eq. 2 in the main text, where the concentrations of the permeate and feed solution were determined by total organic carbon (TOC) analyzer (ANATAILIN, HTY-CT1000B, China). Additionally, the Stokes radius of PEG molecules was calculated based on Eq. S1:

$r_{s}\text{ = 16.73×}\text{10}^{\text{-12}}\text{×}{M_{w}}^{\text{0.557}}$ (S1)

where $M_{w}$ is molecular weight of PEG. The mean pore size of the membranes equals the Stokes radius of the spherical solute with a 50% rejection. The pore size distribution is derived by the following Eq. S2, which was represented by a probability density function:

$\frac{dR(r_{p})}{dr_{p}}=\frac{1}{r_{p}ln\sigma_{p}\sqrt{2\pi}}exp\left[ -\frac{{(lnr_{p}-ln\mu_{p})}^{2}}{{2(ln\sigma_{p})}^{2}} \right]$ (S2)

where μ_p_ is the mean pore size, σ_p_ equals to the ratio of the solute radius at R =84.13% to R =50.00%, and r_p_ is the Stokes radius of the spherical solute.

**S1.3 MD simulations**

The MD simulations were performed using the Gromacs 2022.1 program [S2], conducted under constant temperature and pressure conditions with periodic boundary conditions. A single simulation system, which primarily comprises a COF molecular layer and a plane formed by 20 PEI chains with a degree of polymerization of 20 was built. The distance between the COF and PEI was set at 3.0 nm. Subsequently, in addition to Cl⁻ of COF, this system was filled with 173 Cl⁻ ions, 173 Li⁺ ions, and 17,212 water molecules. The molecules were placed within a cuboid box measuring 9.0 nm × 8.0 nm × 8.0 nm. Both the COF and PEI molecules, along with ions, were modeled using the GAFF all-atom force field, and the TIP3P water model was employed to solvate the complex system [S3]. An initial energy minimization of 50,000 steps was conducted using the steepest descent method. The simulations utilized the NPT ensemble, with the Leap-Frog algorithm used to integrate the equations of motion. Long-range electrostatic interactions were treated using the PME method [S4], while van der Waals and Coulomb interactions were truncated at 12 Å and updated every 10 steps. The Lincs algorithm was applied to constrain all bond lengths [S5], with parameters set to lincs_iter = 1 and lincs_order = 4. The system temperature was raised from 0 K to 298.15 K using the V-rescale temperature coupler [S6]. The Parrinello-Rahman method was employed to maintain a constant pressure of 1 bar [S7], ensuring isotropic pressure conditions. Non-bonded interactions were computed using a neighbor grid-based cutoff scheme, with short and long cutoff distances set at 9 Å and 14 Å, respectively. All simulations were initialized with velocities randomly assigned according to the Maxwell-Boltzmann distribution, with a total of 50,000,000 steps simulated at a time step of 1 fs, resulting in a total simulation time of 50 ns, generating 2000 conformations. Visualization of simulation results was achieved using the built-in Gromacs tools and VMD software.

**S1.4 DFT calculations**

The interaction energies quantitatively reveal the distinct roles of COF nanosheets and PEI. The first principles density-functional theory calculations were carried out using the DMol3 module in Materials Studio. The electron exchange–functional was calculated using Perdew-Burke–Ernzerhof (PBE) described by generalized gradient approximation (GGA). An all electron double numerical atomic orbital augmented by Double Numerical plus polarization (DNP) is used as the basis set and all electrons are included in the calculation. The convergence criteria in total energy, maximum force, and maximum displacement were set at 10^-5^ Hartree, 0.002 Hartree/Å, and 0.005 Å, respectively. The electronic self-consistent field (SCF) tolerance was set at 10^-6^ Hartree. Accordingly, the interaction energy is defined as follows:

|  | *E(A-B)=E(total)－E(A)－E(B)* | (S3) |
| --- | --- | --- |

where *E(A-B)* is the interaction energy between Li^+^/Cl^-^ and COF/PEI; E(total) is the energy of Li^+^/Cl^-^ and COF/PEI; E(A) is the energy of Li^+^ or Cl^-^ and E(B) is the energy of COF or PEI, respectively.


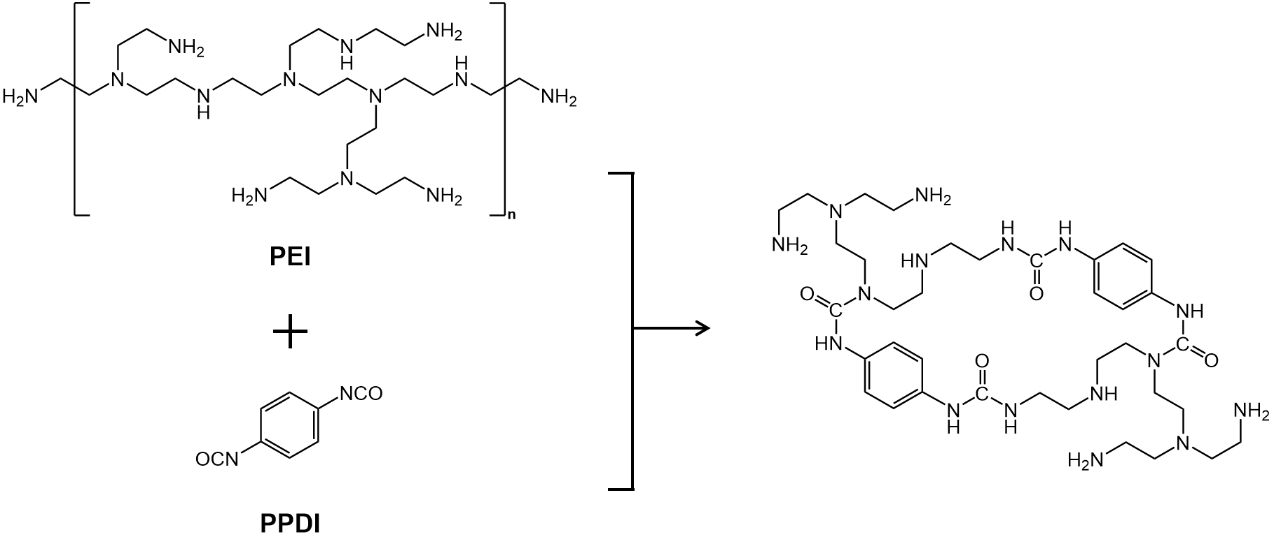


**Scheme S2** Schematic illustration of the structure of polyuria

**S2 Supplementary Figures and Tables**


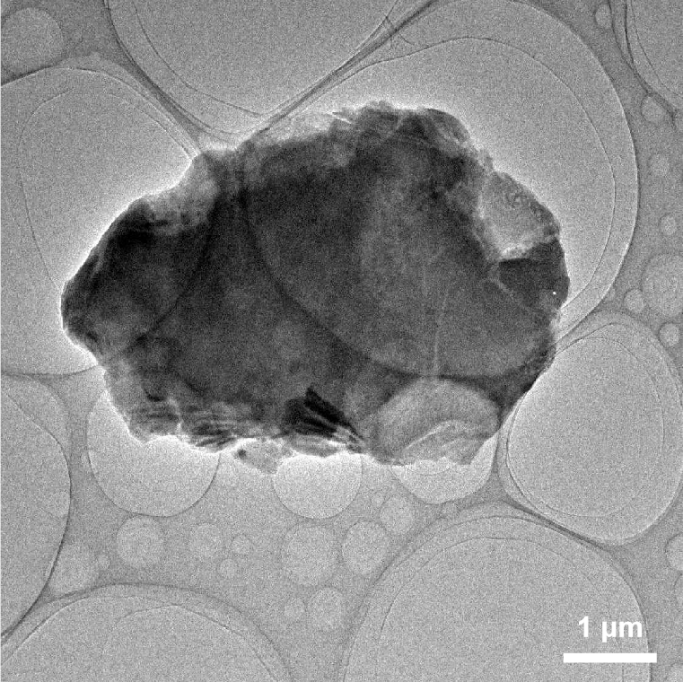


**Fig. S1** TEM image of COF nanosheets


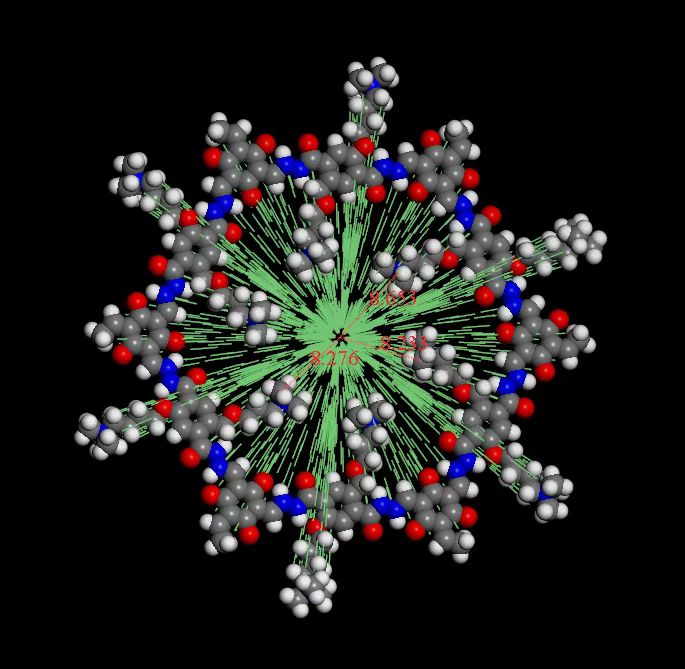


**Fig. S2** Pore size of COF

The pore size was simulated via Material Studio.


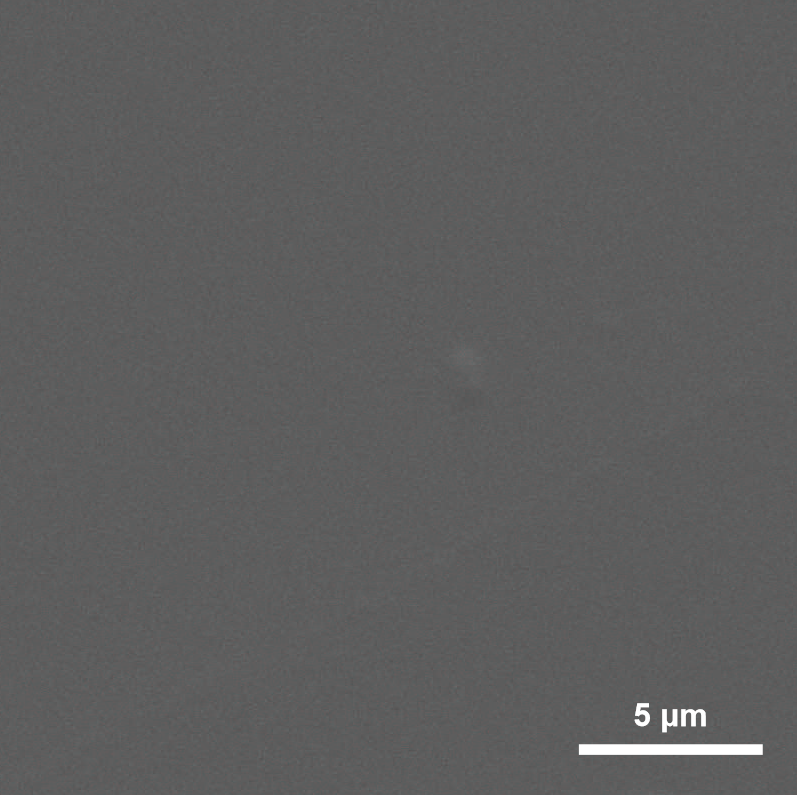


**Fig. S3** SEM image of COF scaffold membranes


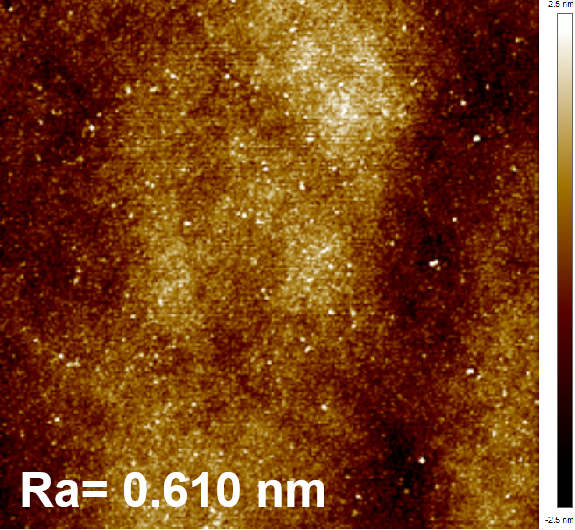


**Fig. S4** AFM image of COF scaffold membranes


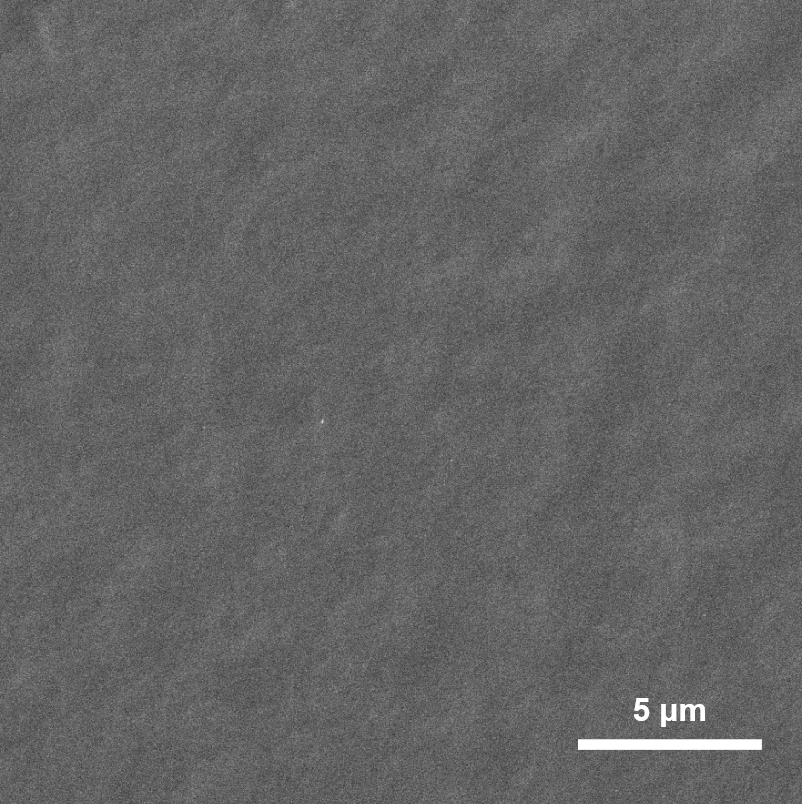


**Fig. S5** SEM image of PU membranes


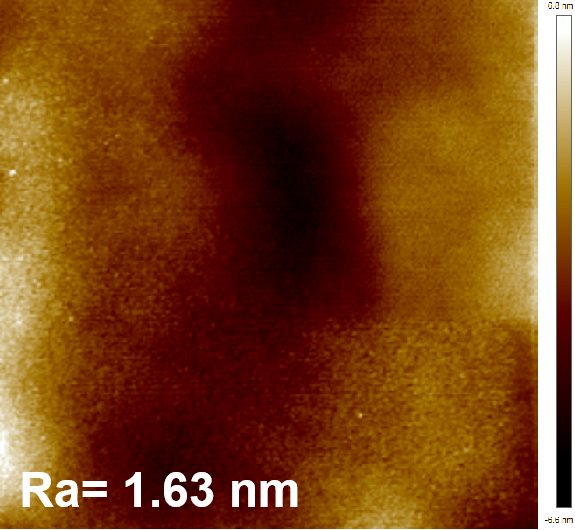


**Fig. S6** AFM image of PU membranes


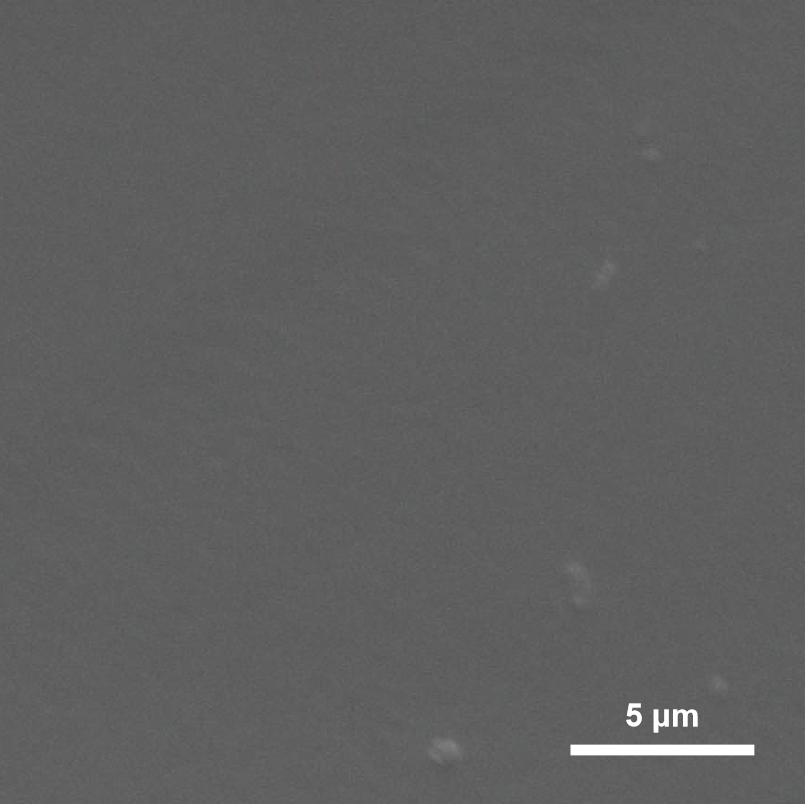


**Fig. S7** SEM image of COF hybrid membranes


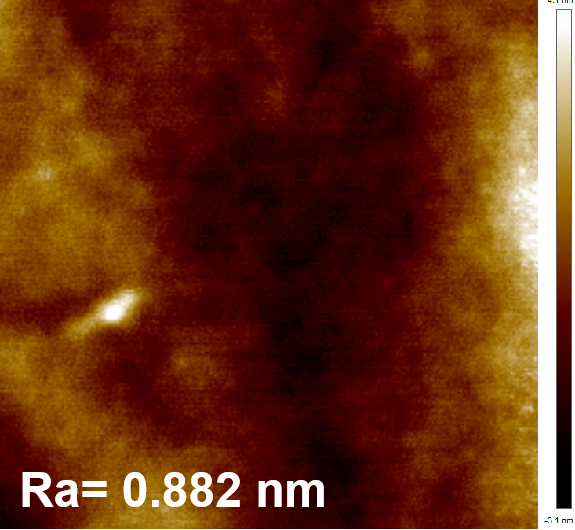


**Fig. S8** AFM image of COF hybrid membranes


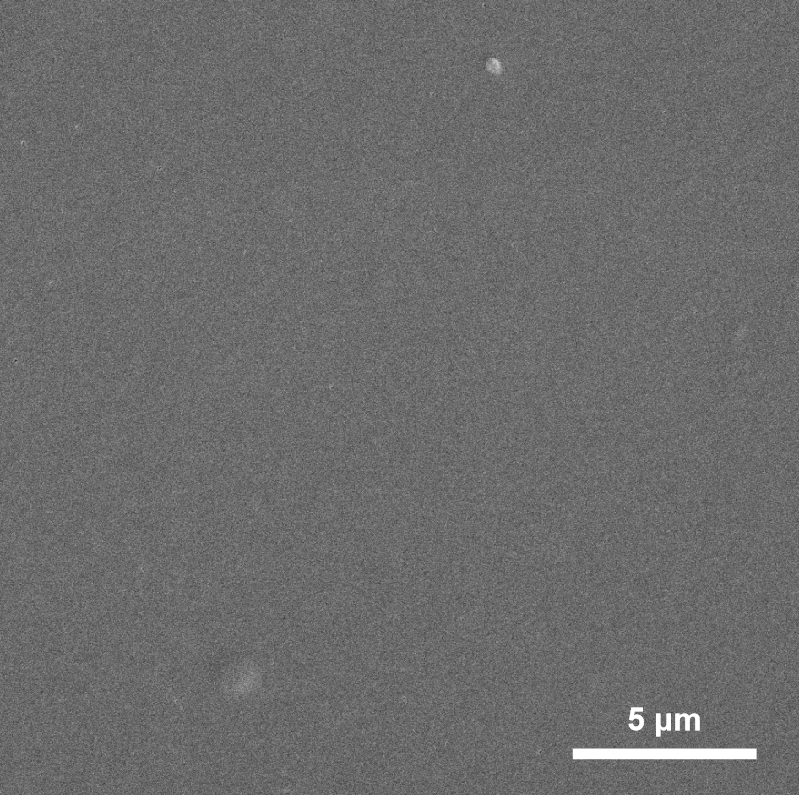


**Fig. S9** SEM image of COF membranes


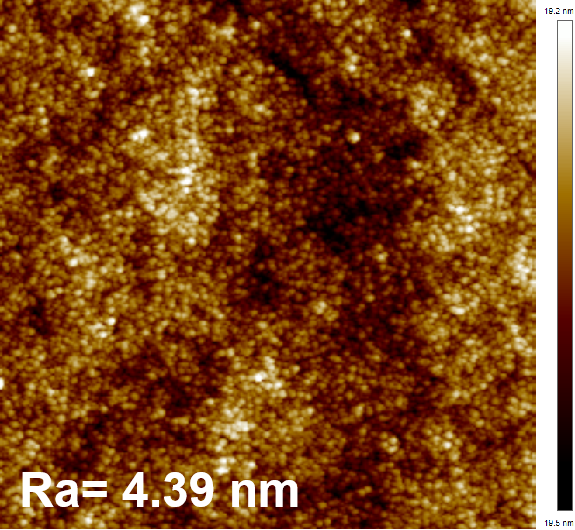


**Fig. S10** AFM image of COF membranes


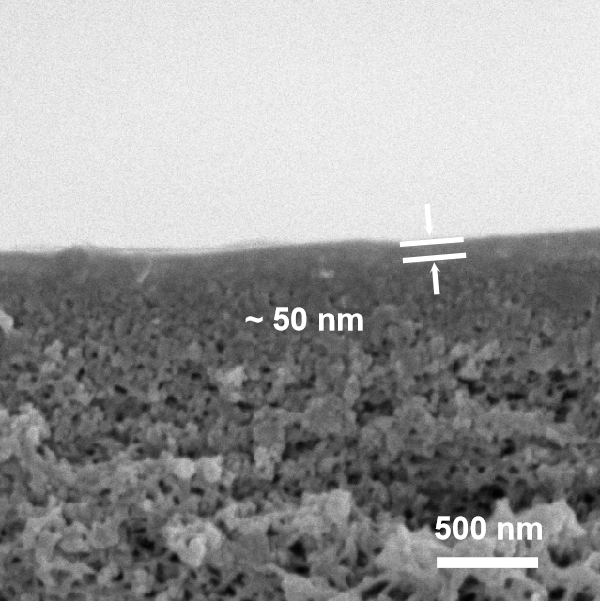


**Fig. S11** Cross-sectional SEM image of PU membranes


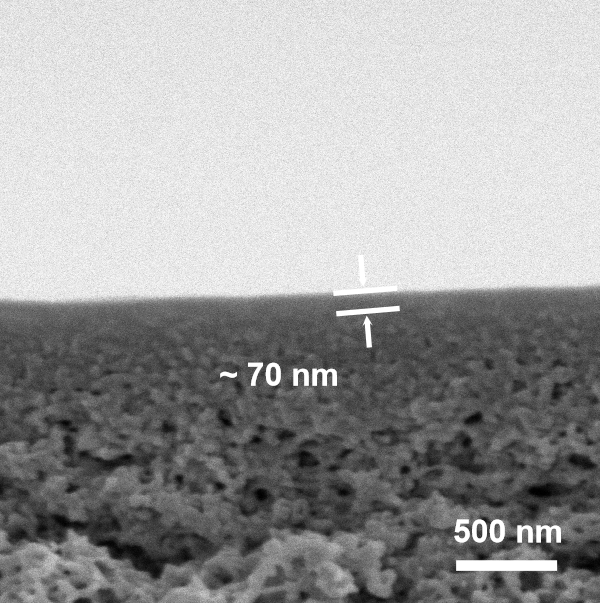


**Fig. S12** Cross-sectional SEM image of COF hybrid membranes


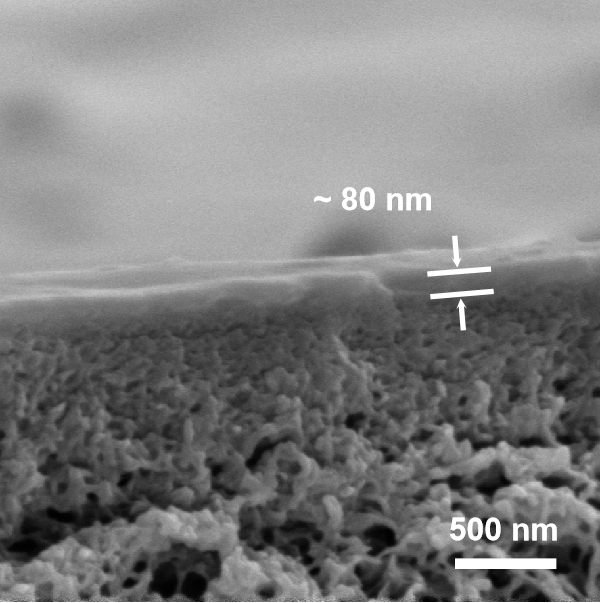


**Fig. S13** Cross-sectional SEM image of COF membranes


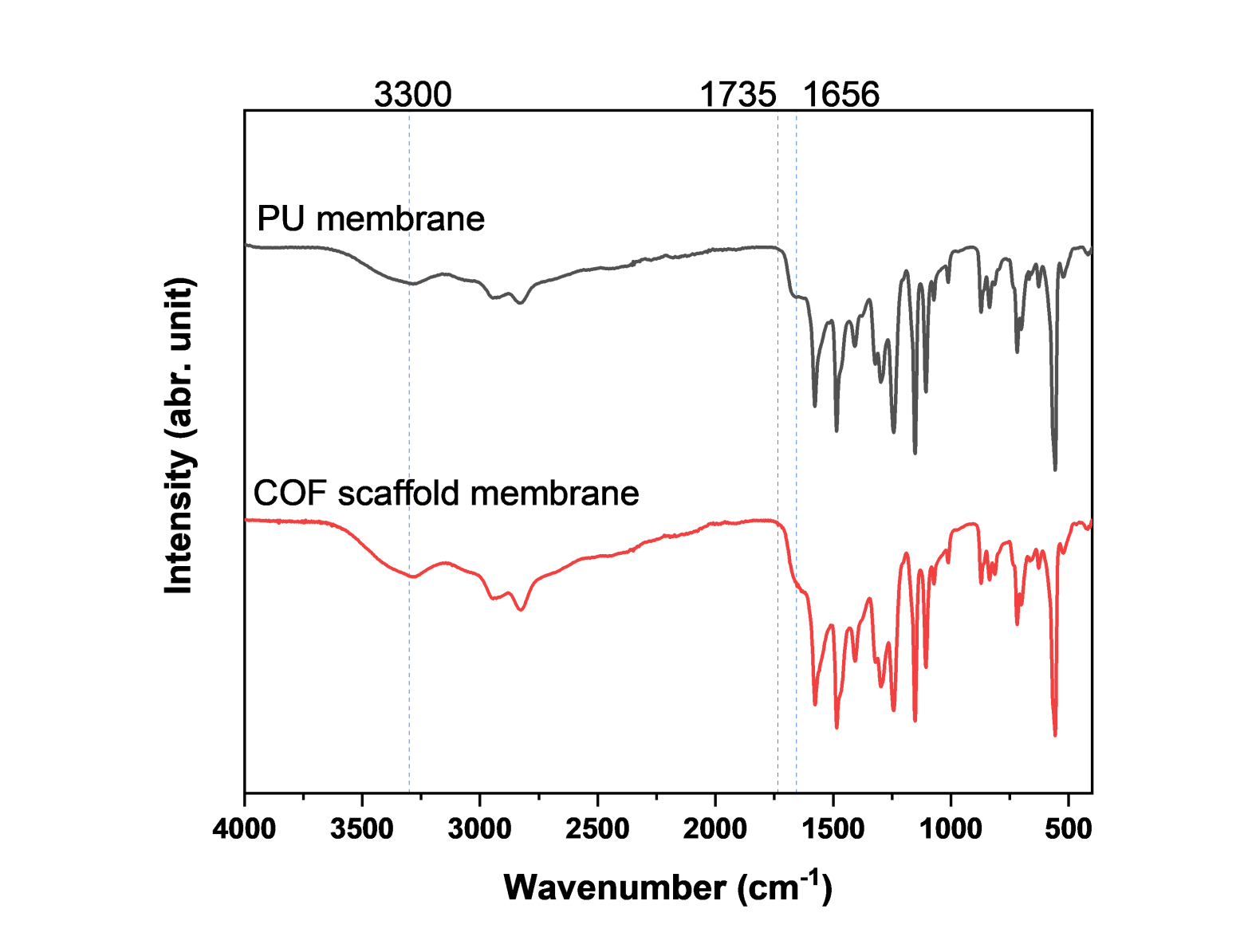


**Fig. S14** FTIR spectra of COF scaffold membranes


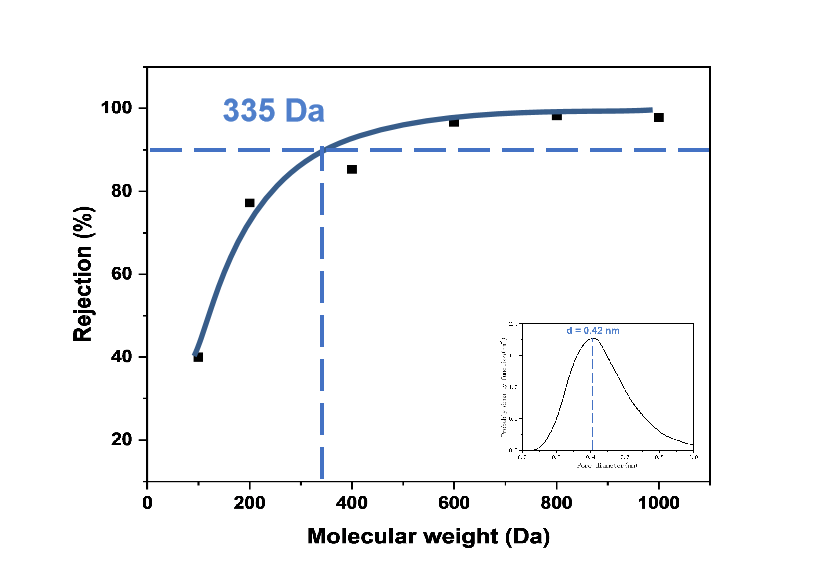


**Fig. S15** MWCO of COF scaffold membranes


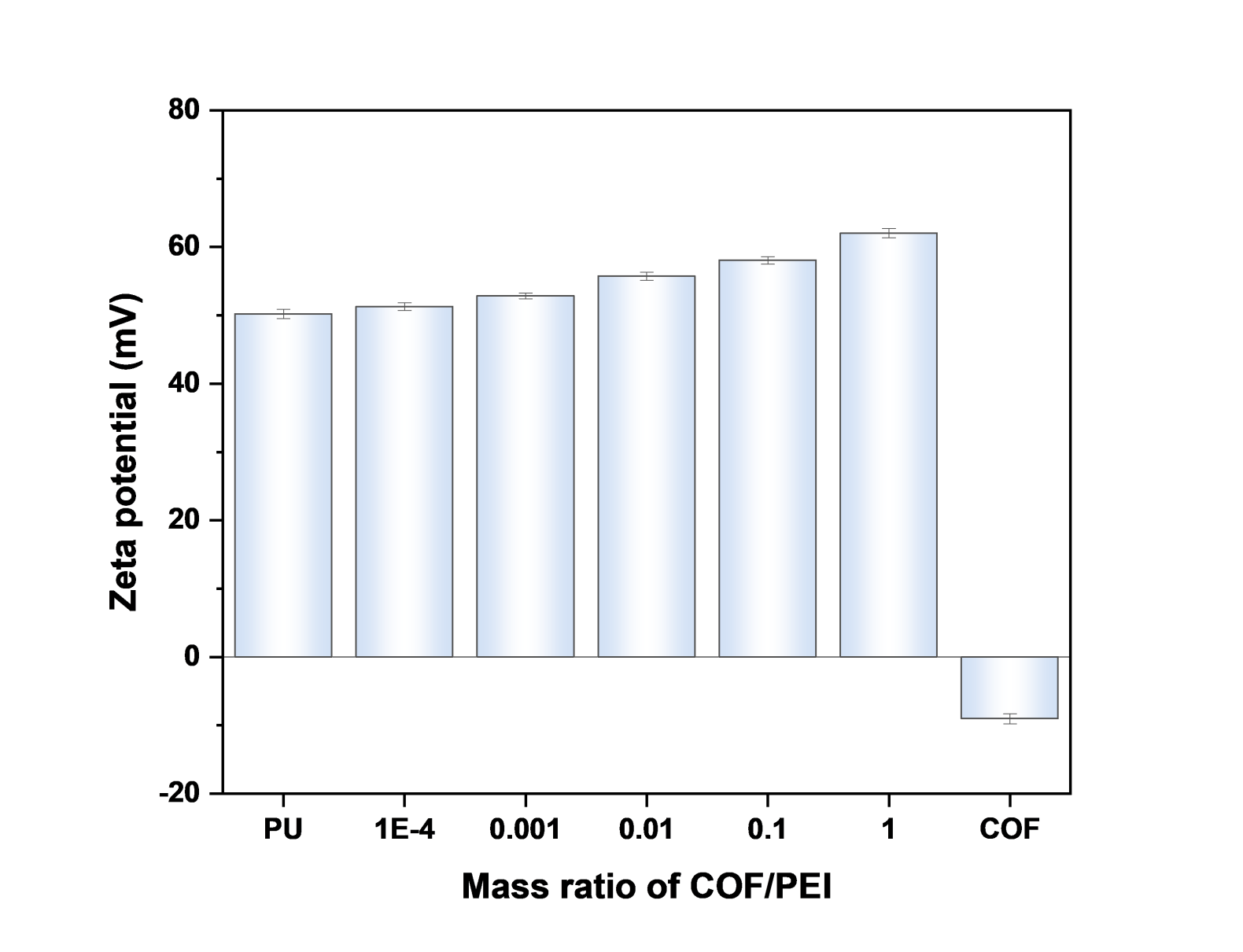


**Fig. S16** Effect of mass ratio of COF/PEI on zeta potential of COF scaffold membranes


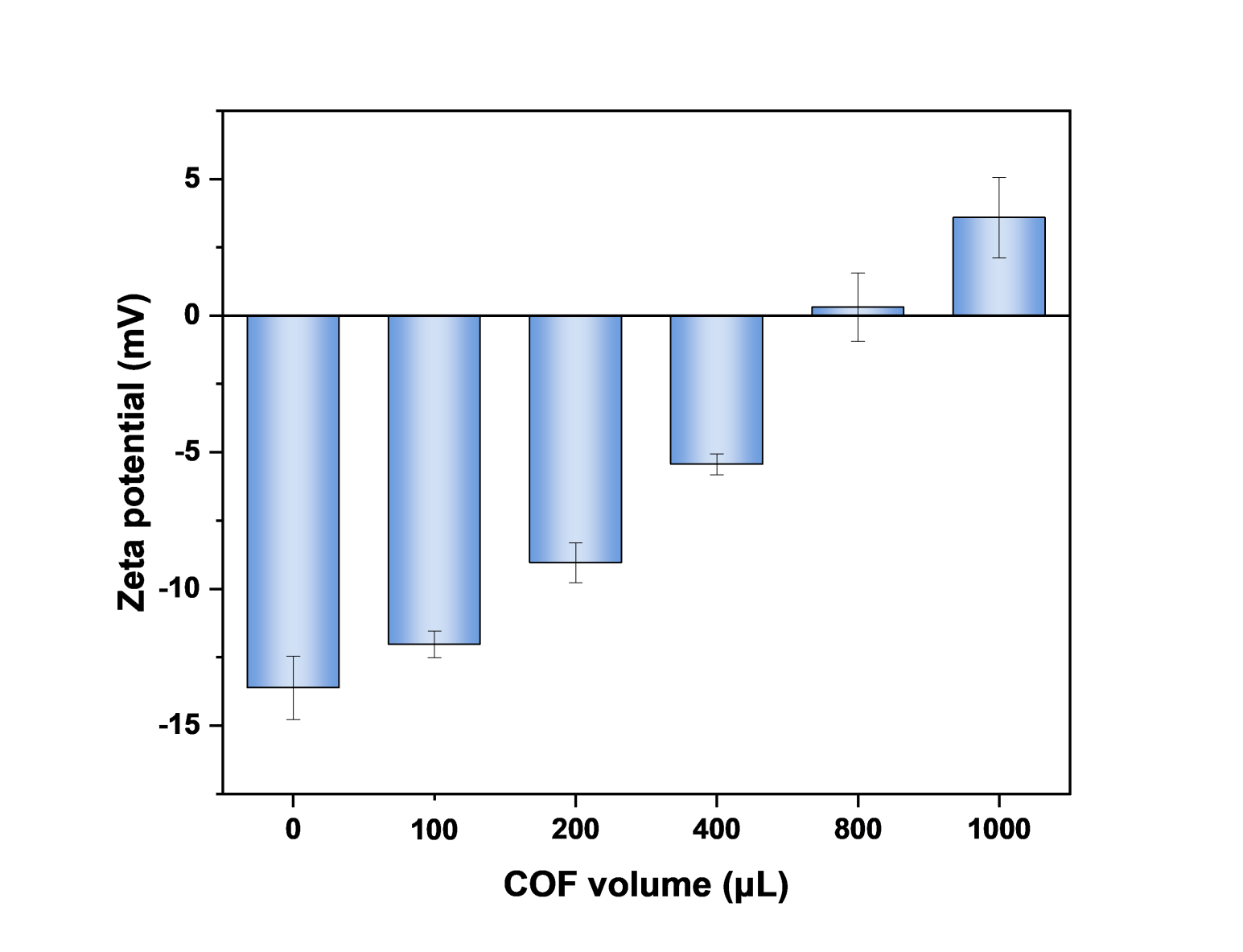


**Fig. S17** Effect of COF volume on zeta potential of COF membranes


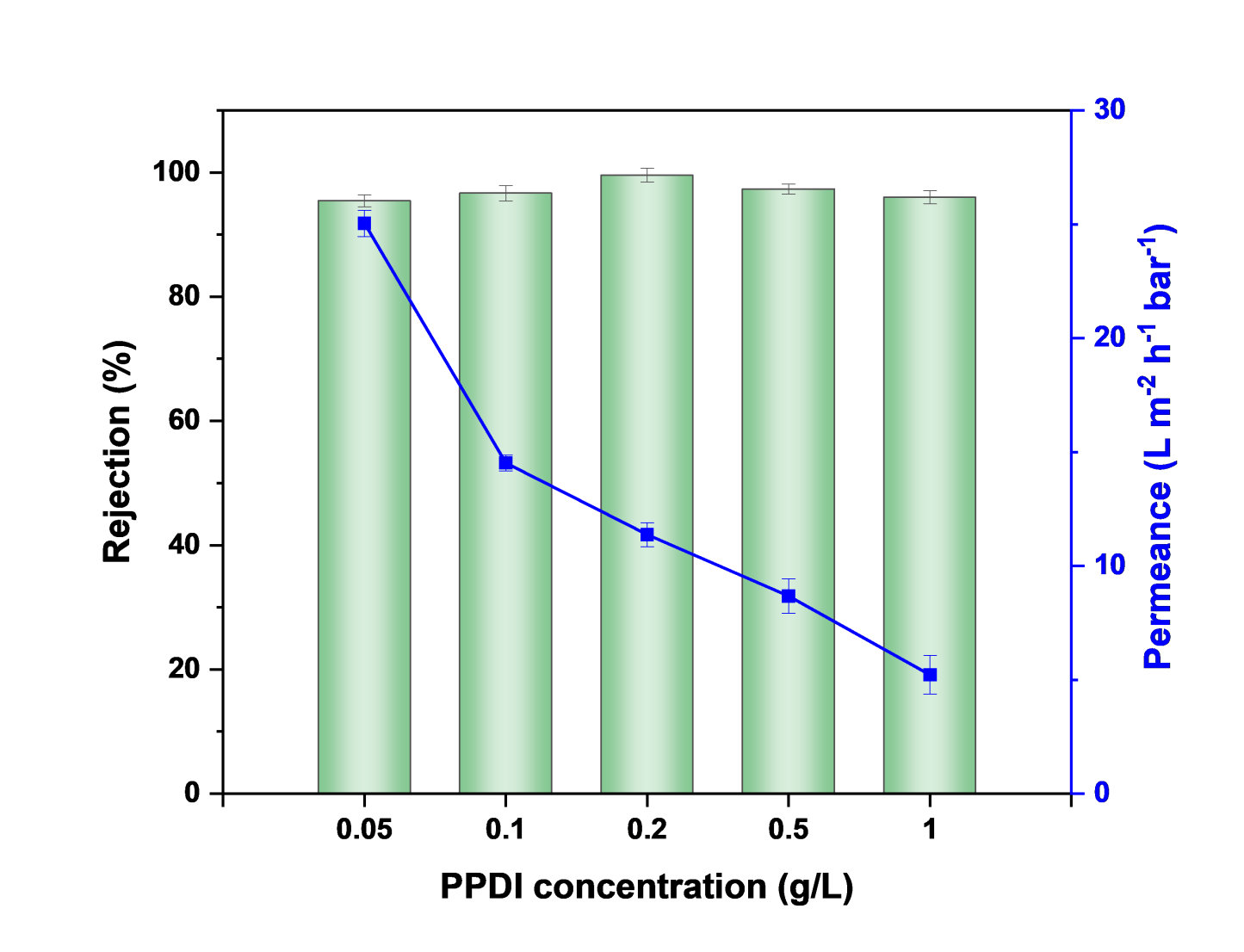


**Fig. S18** Effect of PPDI concentration on performance of COF scaffold membranes


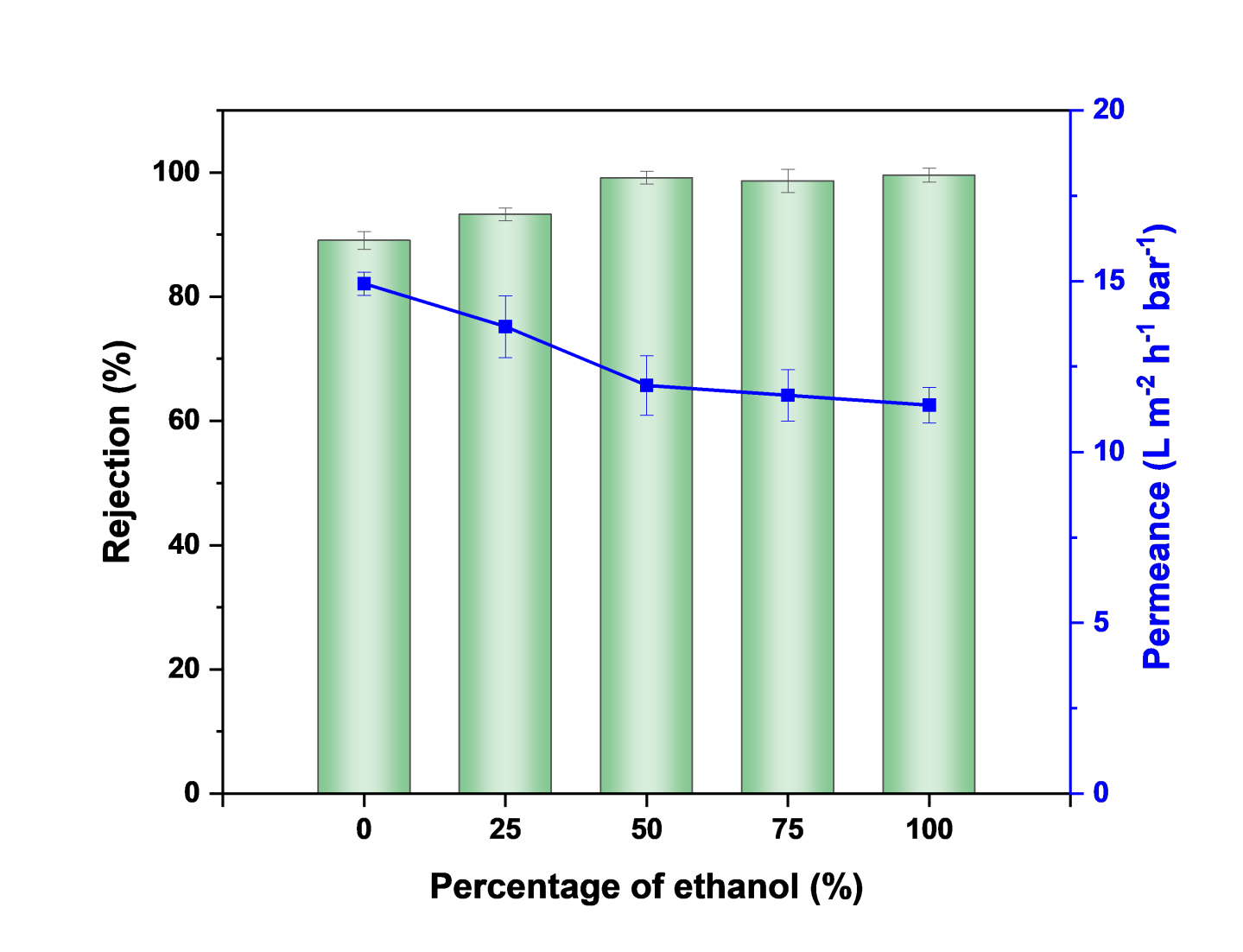


**Fig. S19** Effect of volume ratio of water/ethanol on performance of COF scaffold membranes


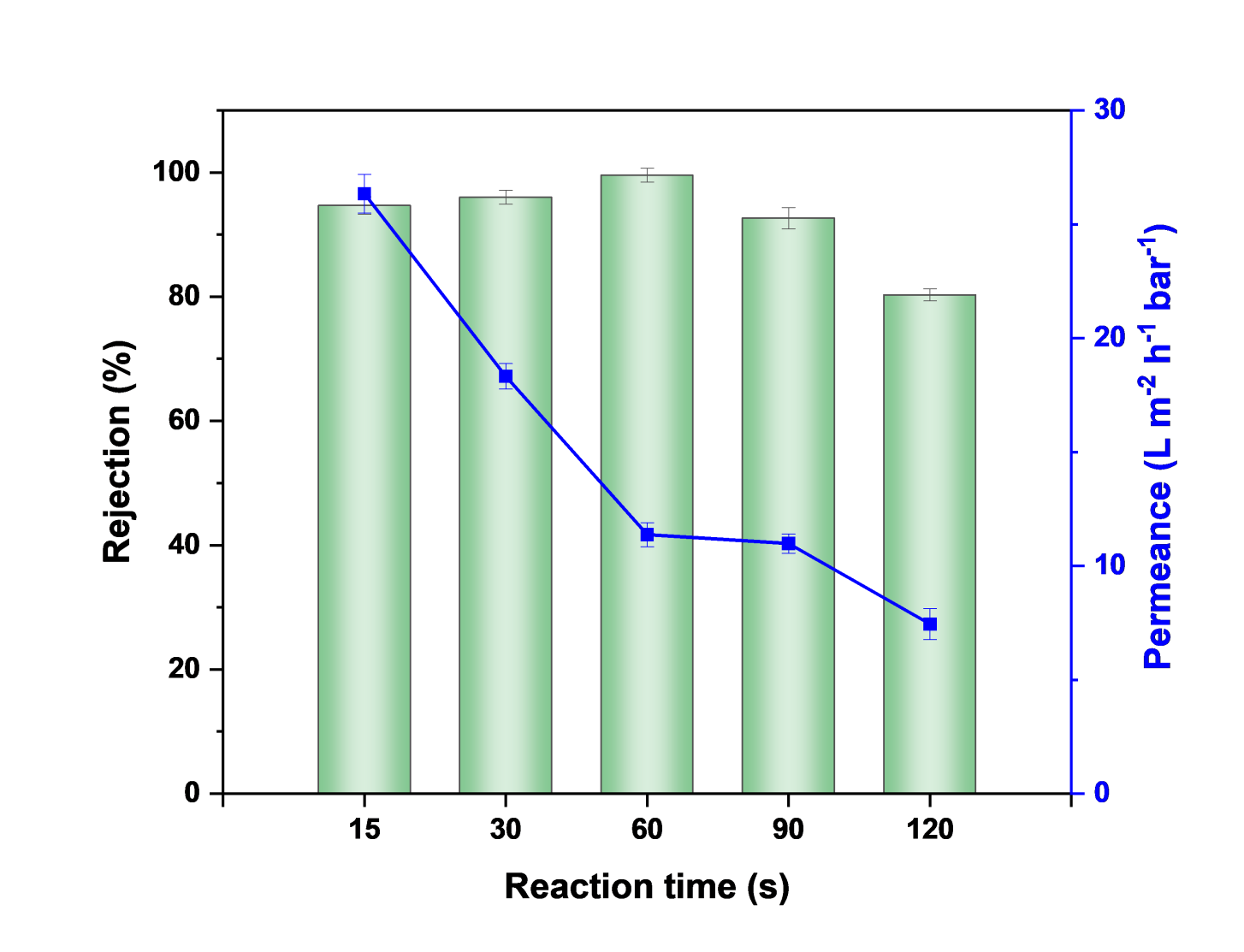


**Fig. S20** Effect of reaction time on performance of COF scaffold membranes


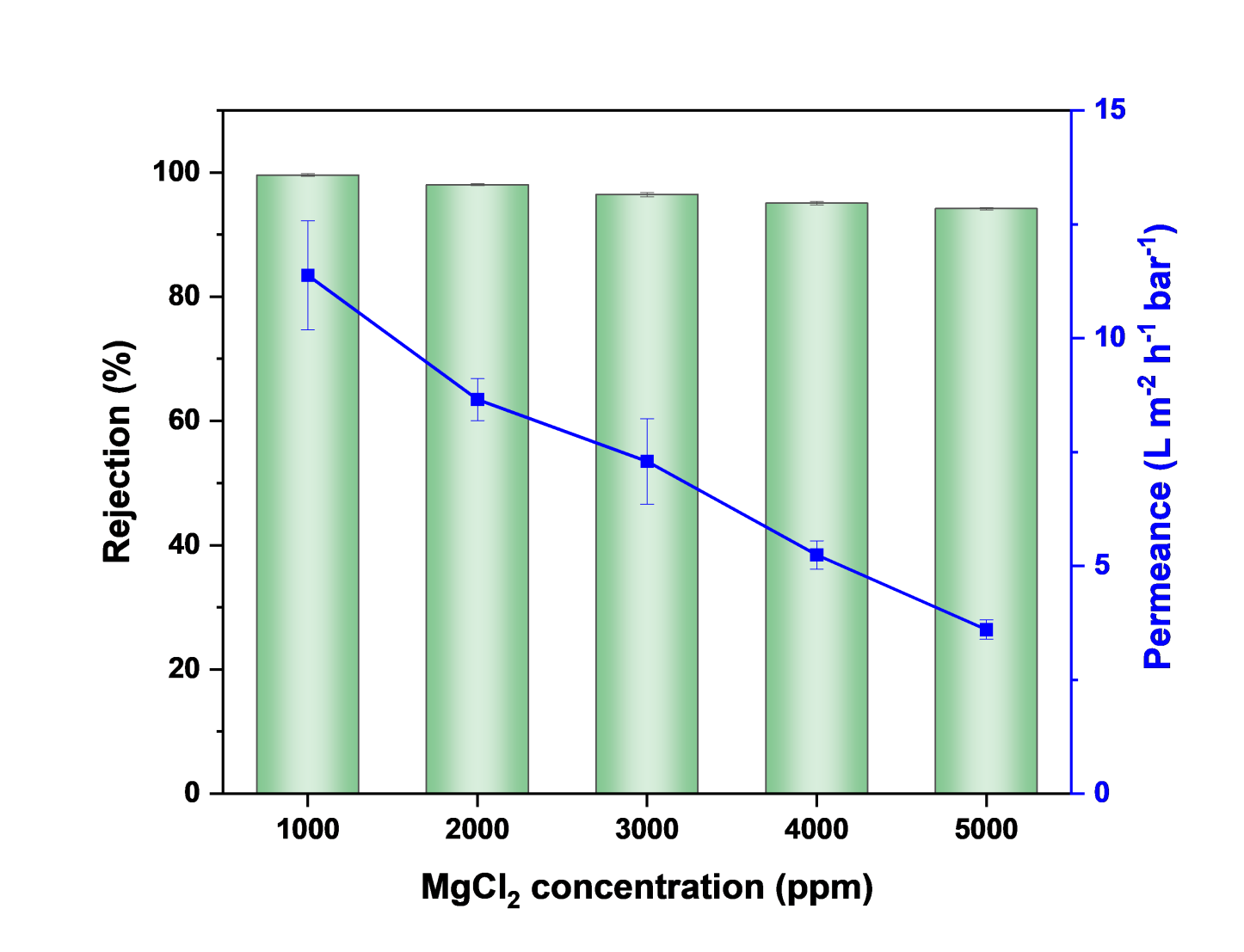


**Fig. S21** Rejection and permeance to 1000 ppm MgCl_2_ with respect to different feed solution concentration


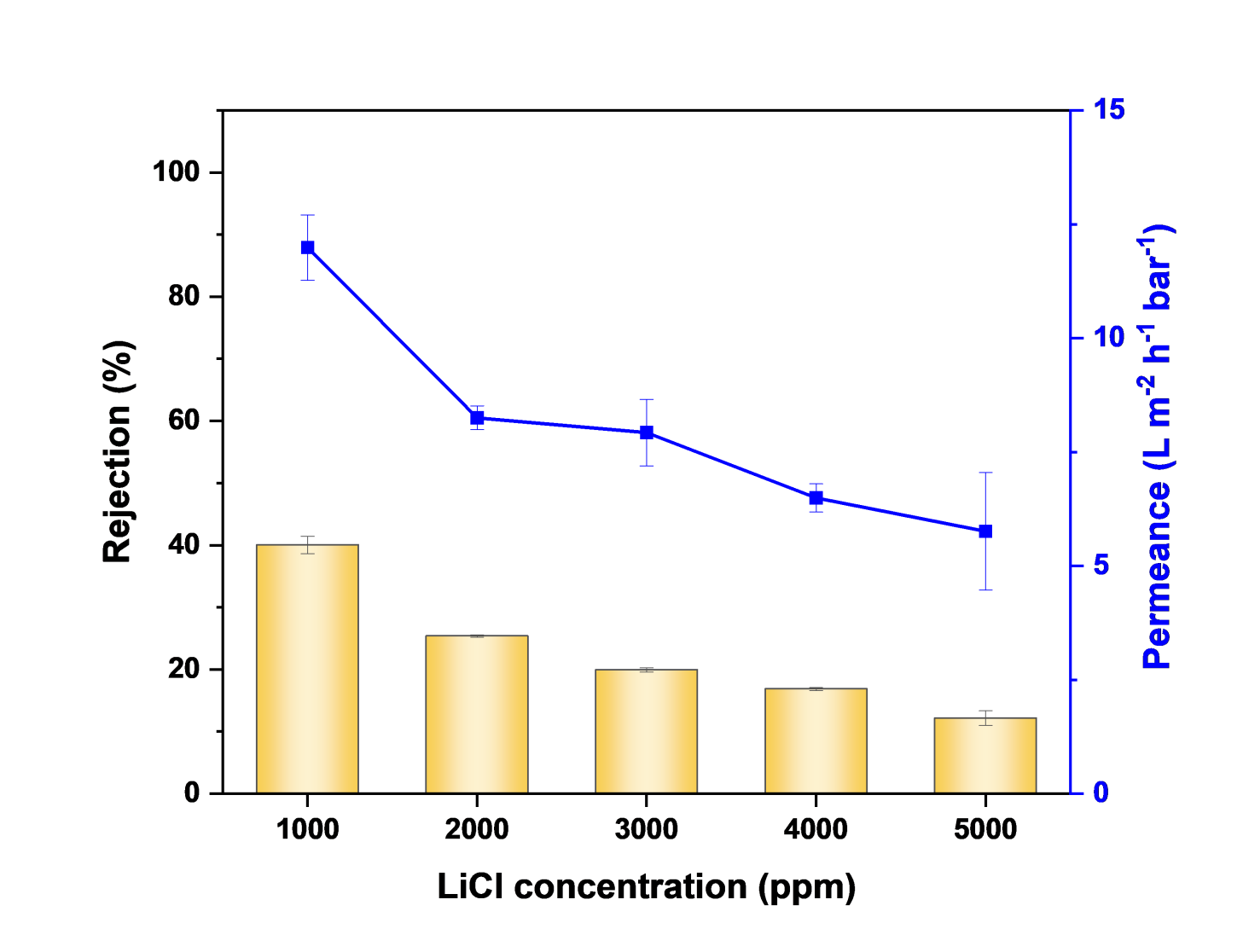


**Fig. S22** Rejection and permeance to 1000 ppm LiCl with respect to different feed solution concentration


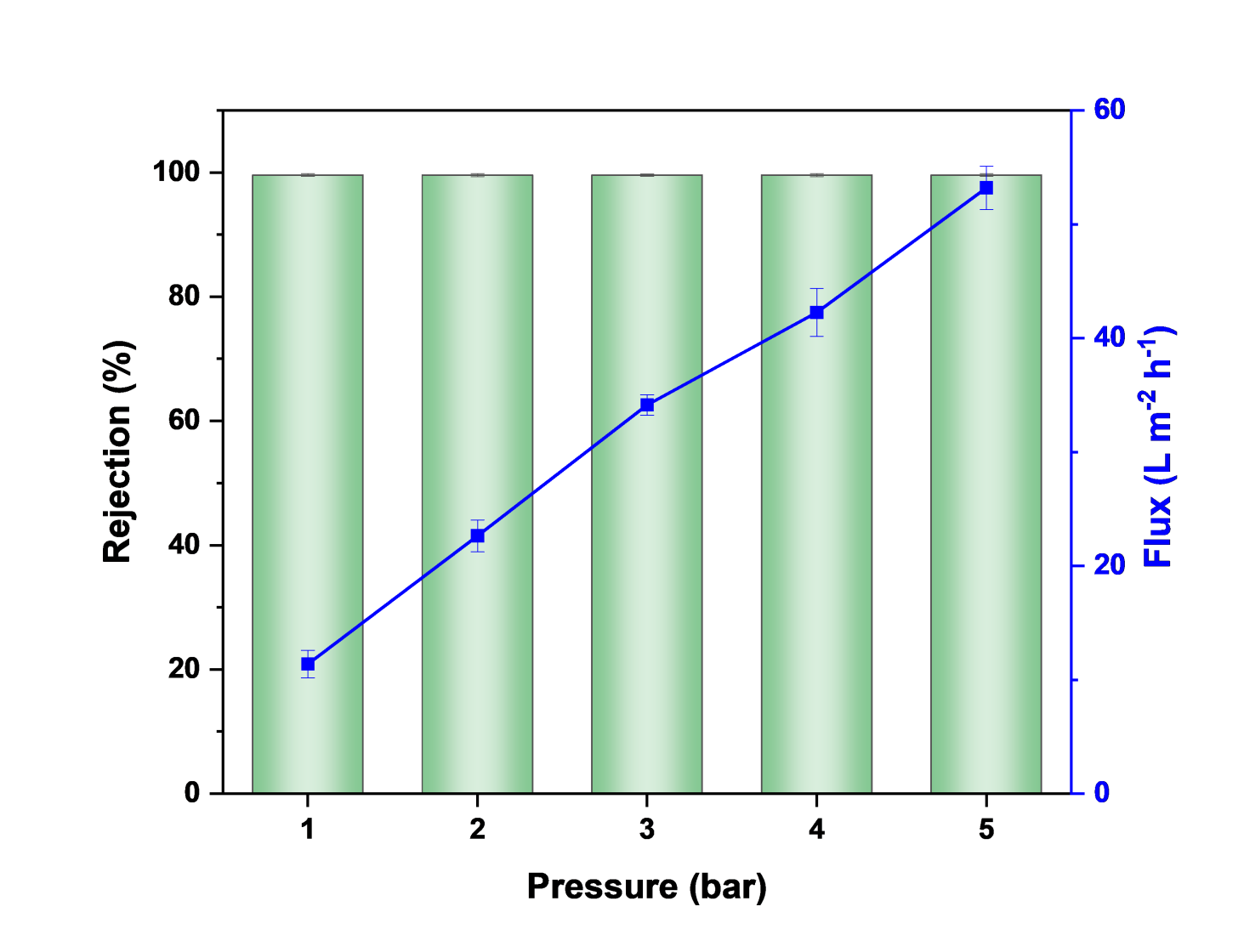


**Fig. S23** Rejection to 1000 ppm MgCl_2_ with different pressure


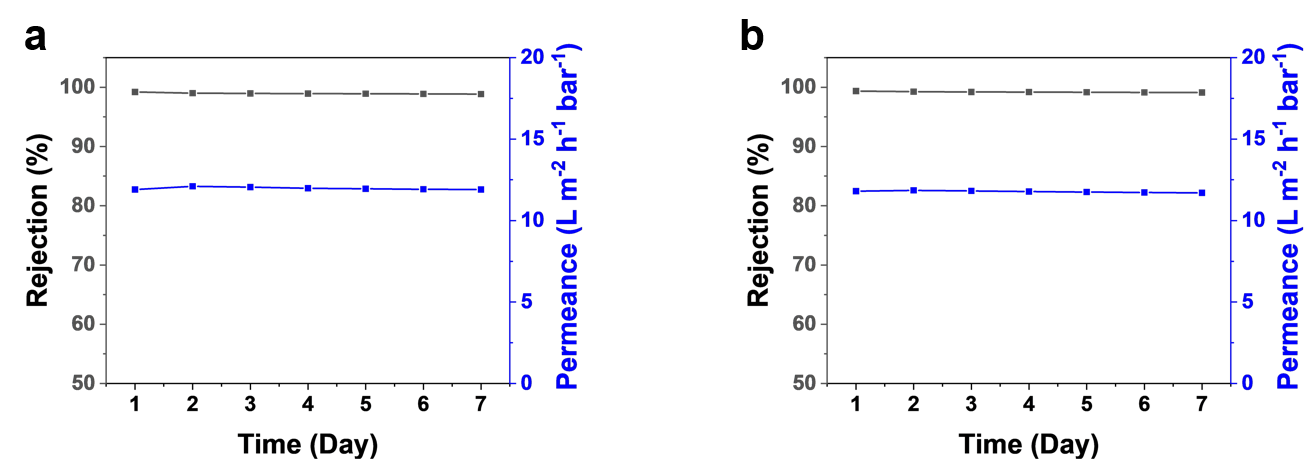


**Fig. S24** The COF scaffold membrane resistant to (a) H_2_SO_4_ (pH=3) and (b) NaOH (pH=11)


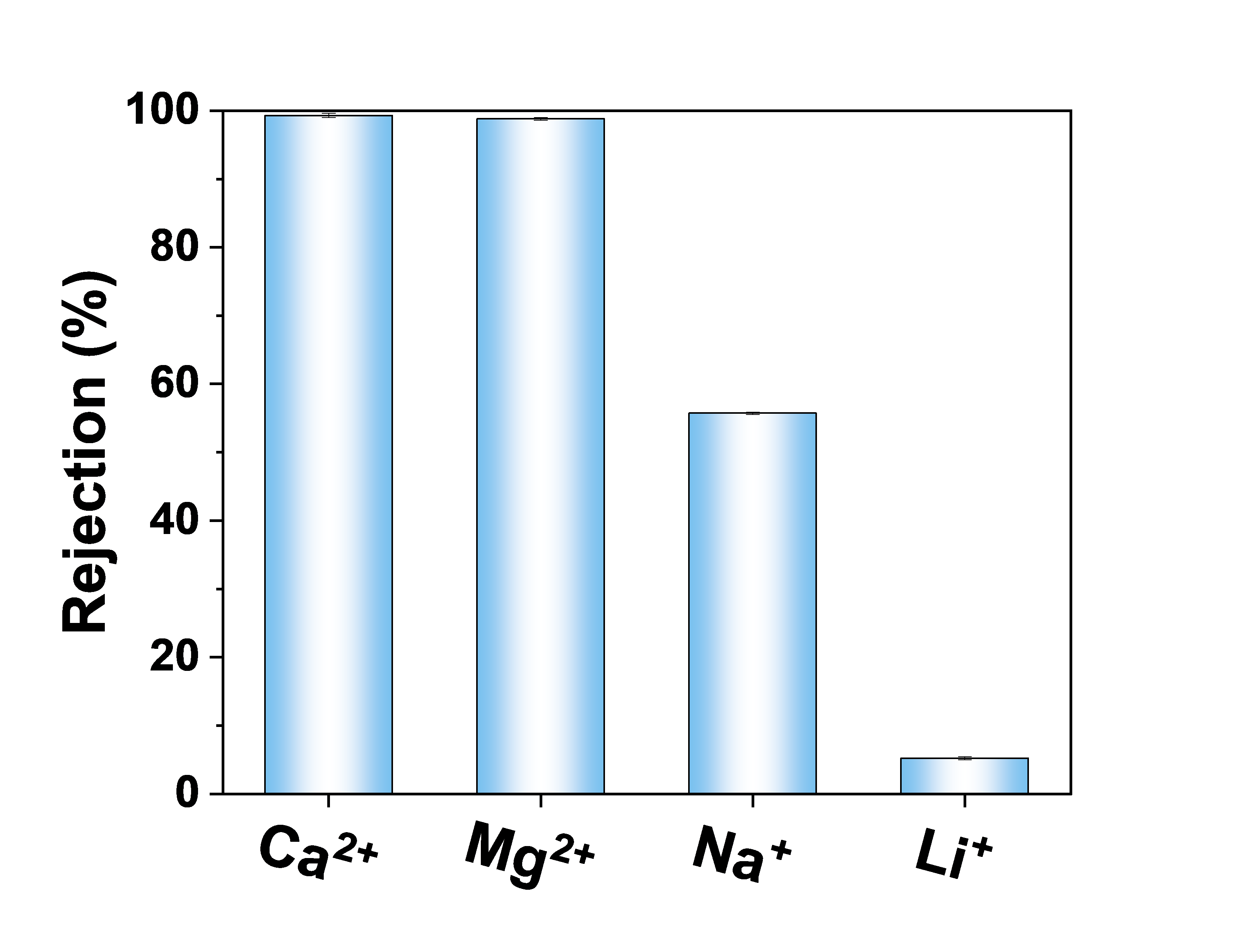


**Fig. S25** Rejection to different salts of simulated salt lake brine


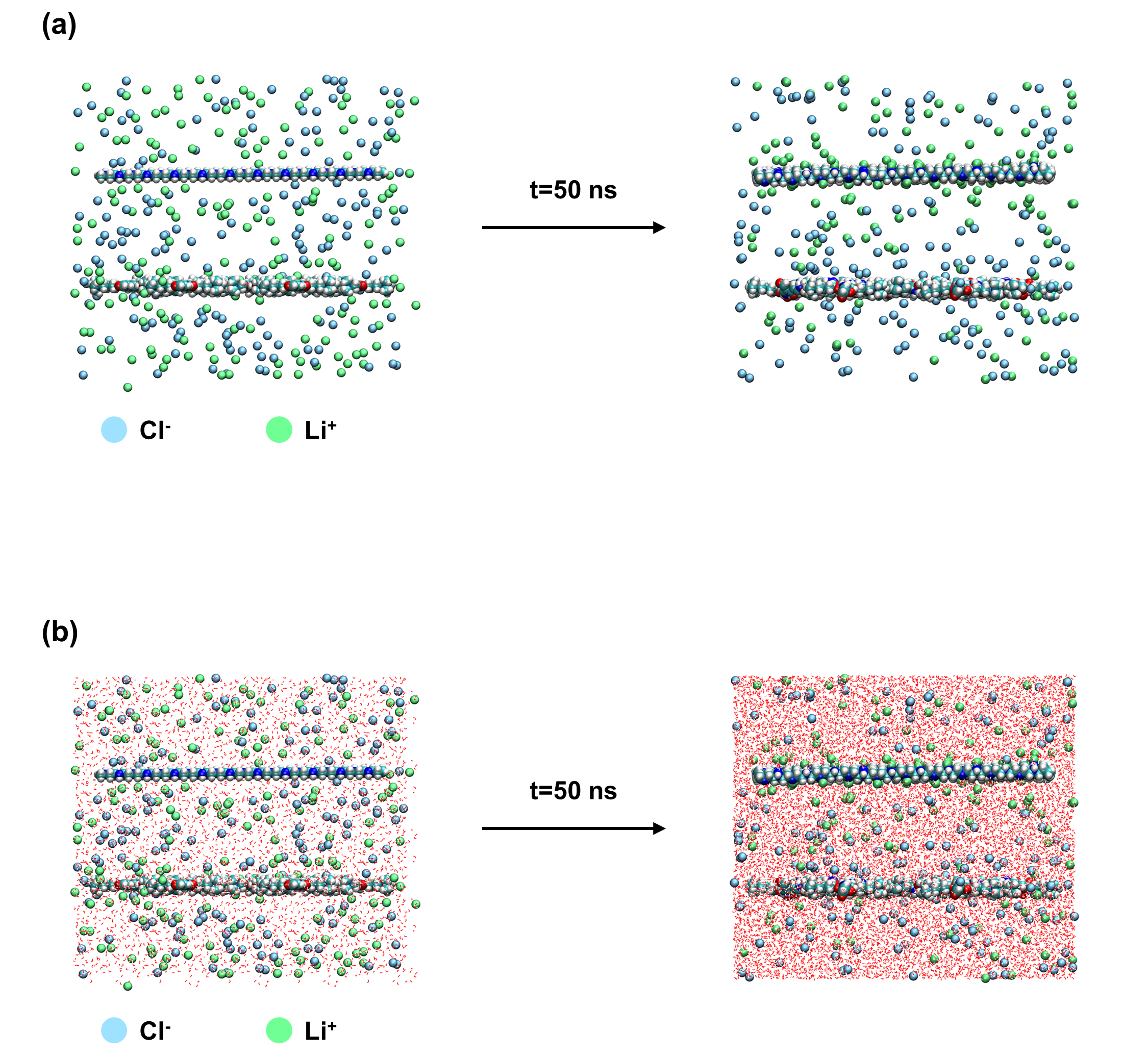


**Fig. S26** MD model without solvent water molecules


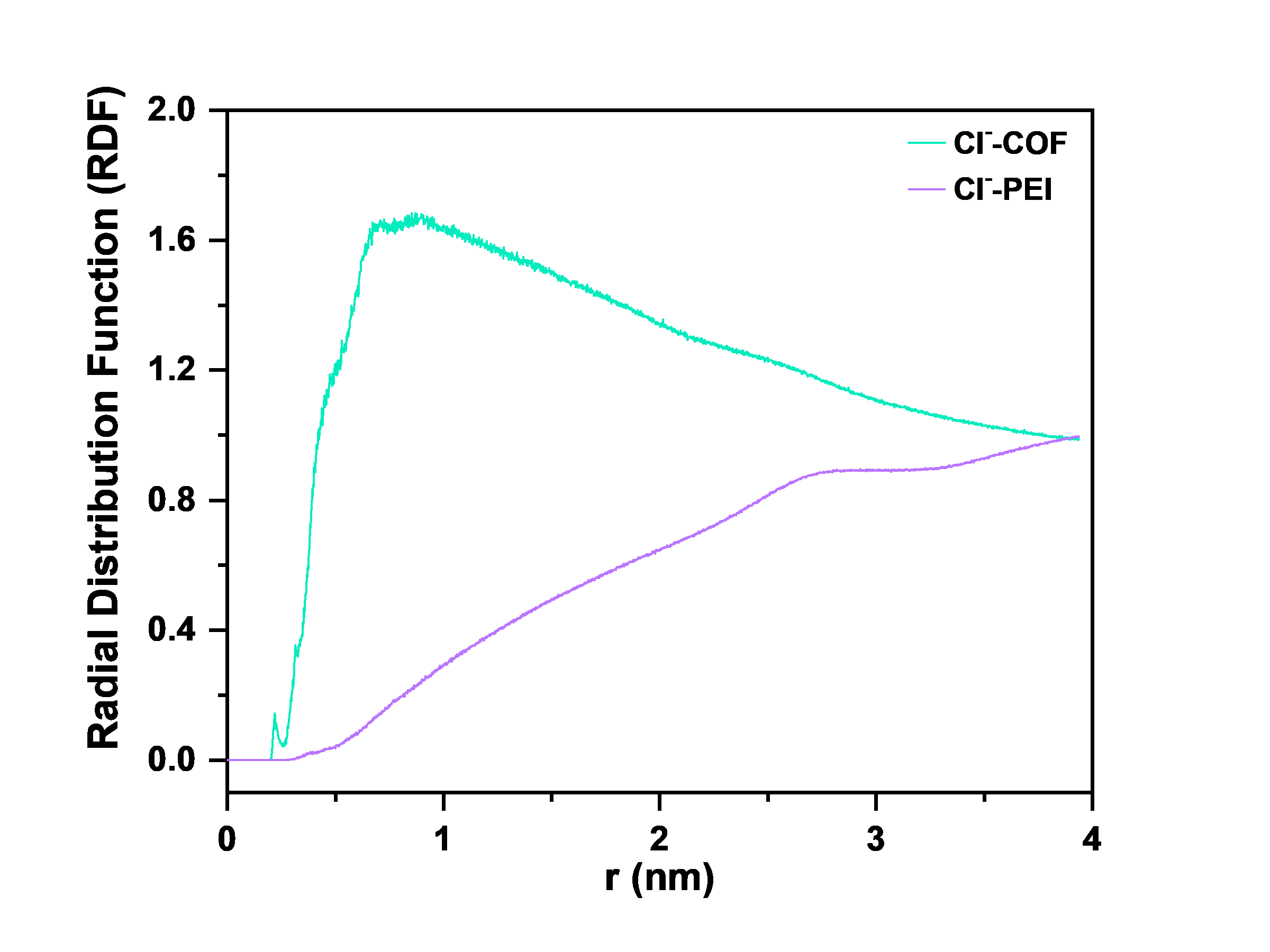


**Fig. S27** RDF distribution of Cl⁻ between COF nanosheets and PEI


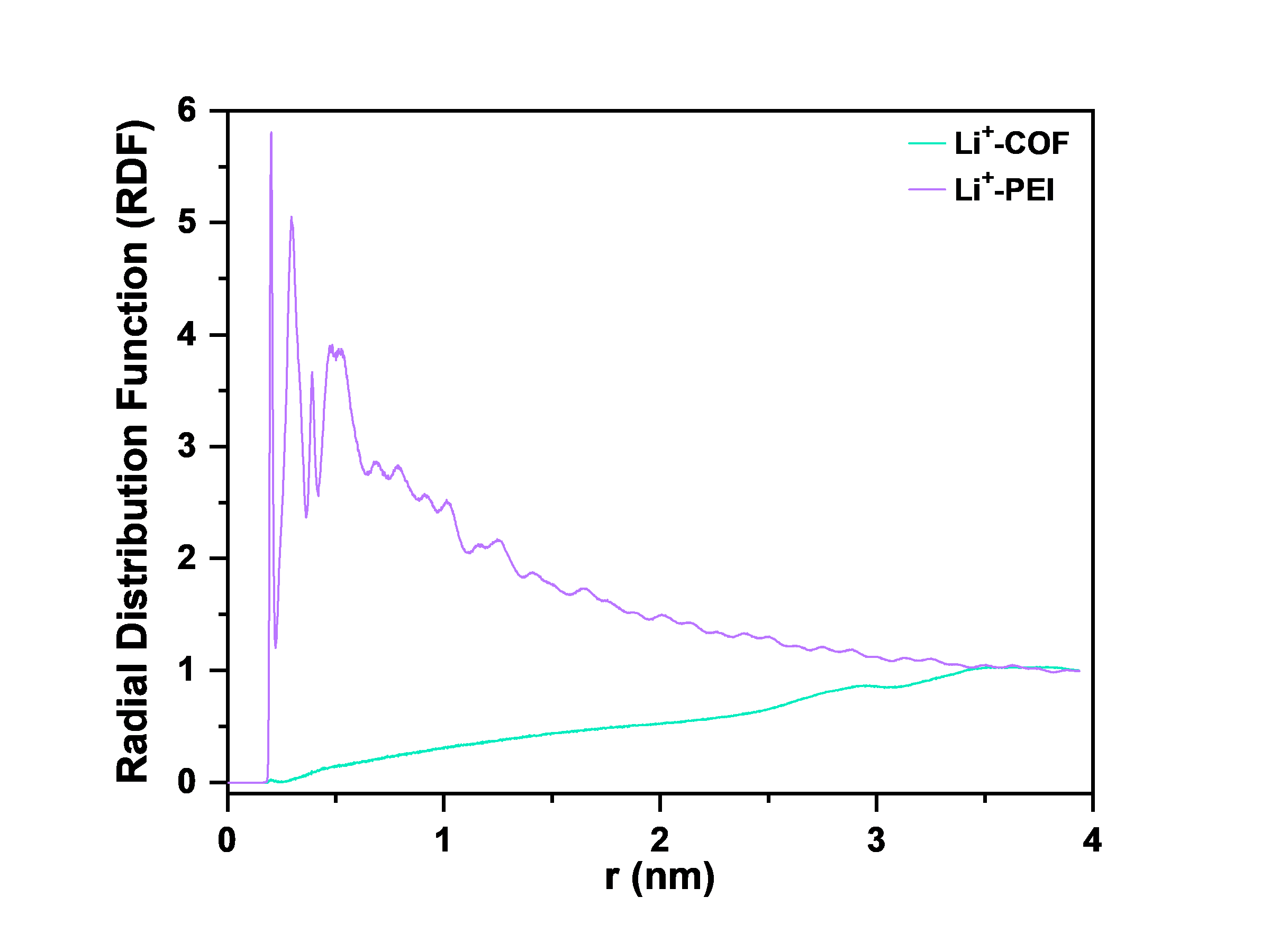


**Fig. S28** RDF distribution of Li⁺ between COF nanosheets and PEI


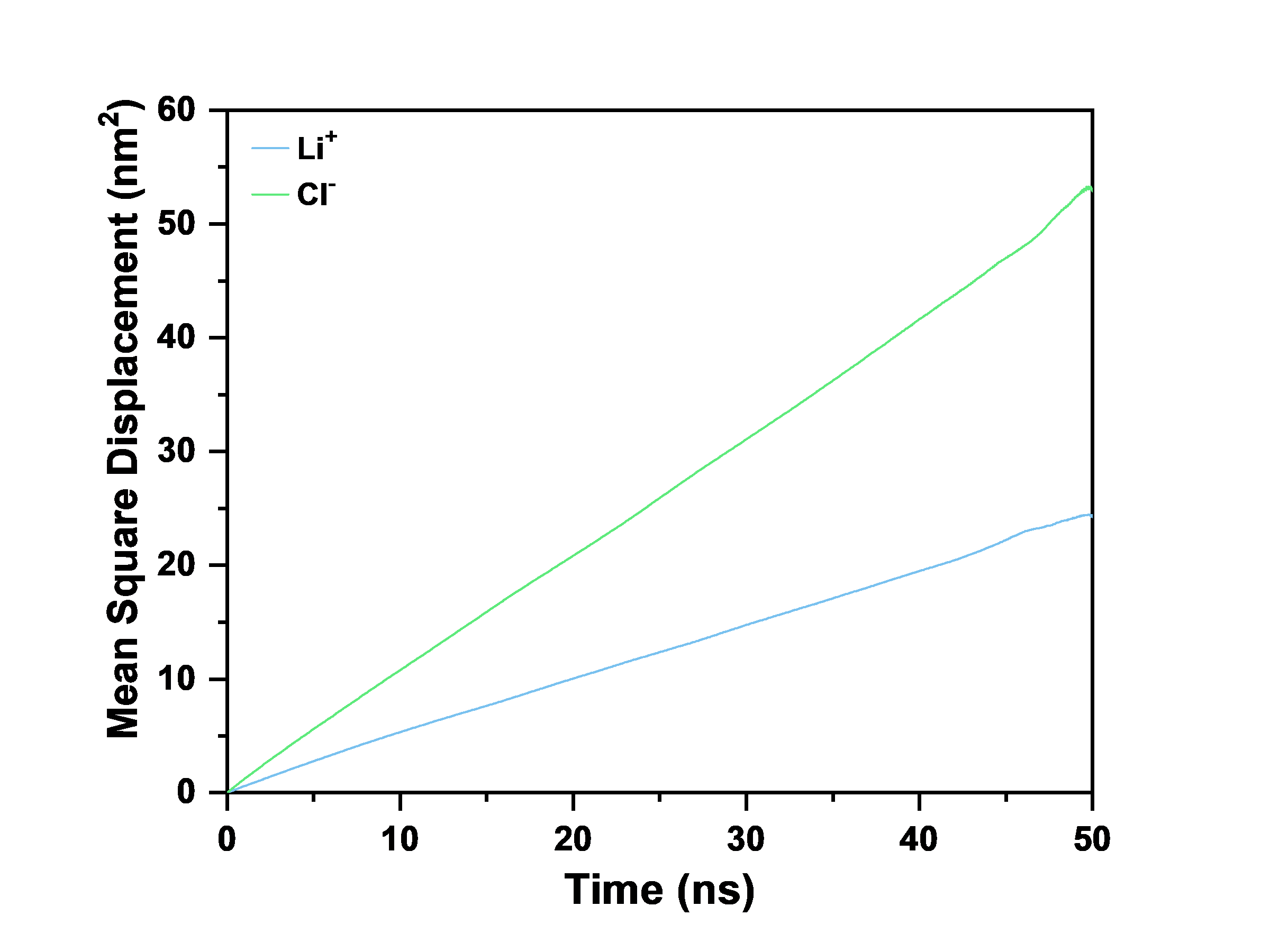


**Fig. S29** MSD of Cl⁻ and Li⁺


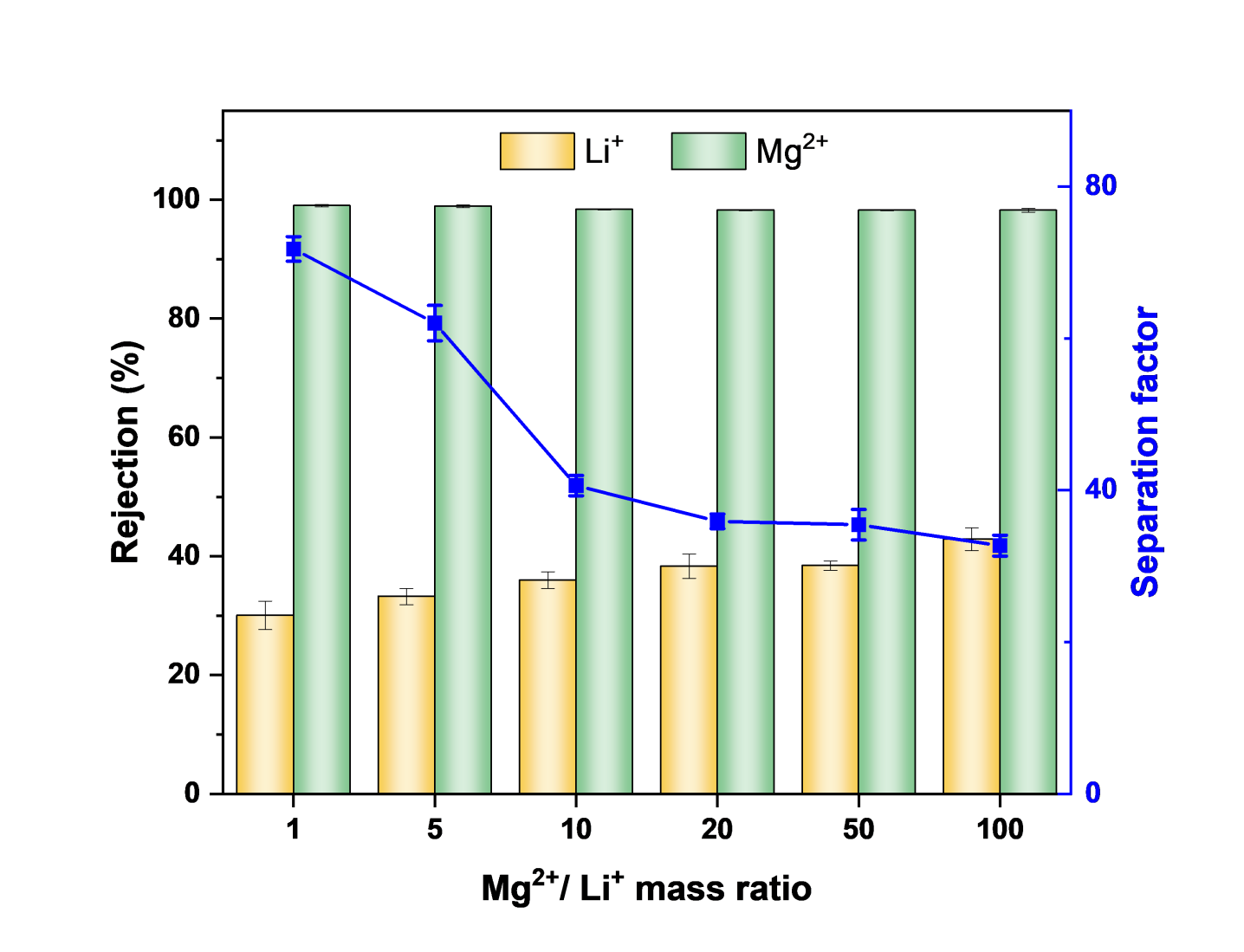


**Fig. S30** Rejection and separation factor of COF scaffold membranes in the single-solute system (MgSO_4_ and Li_2_SO_4_)


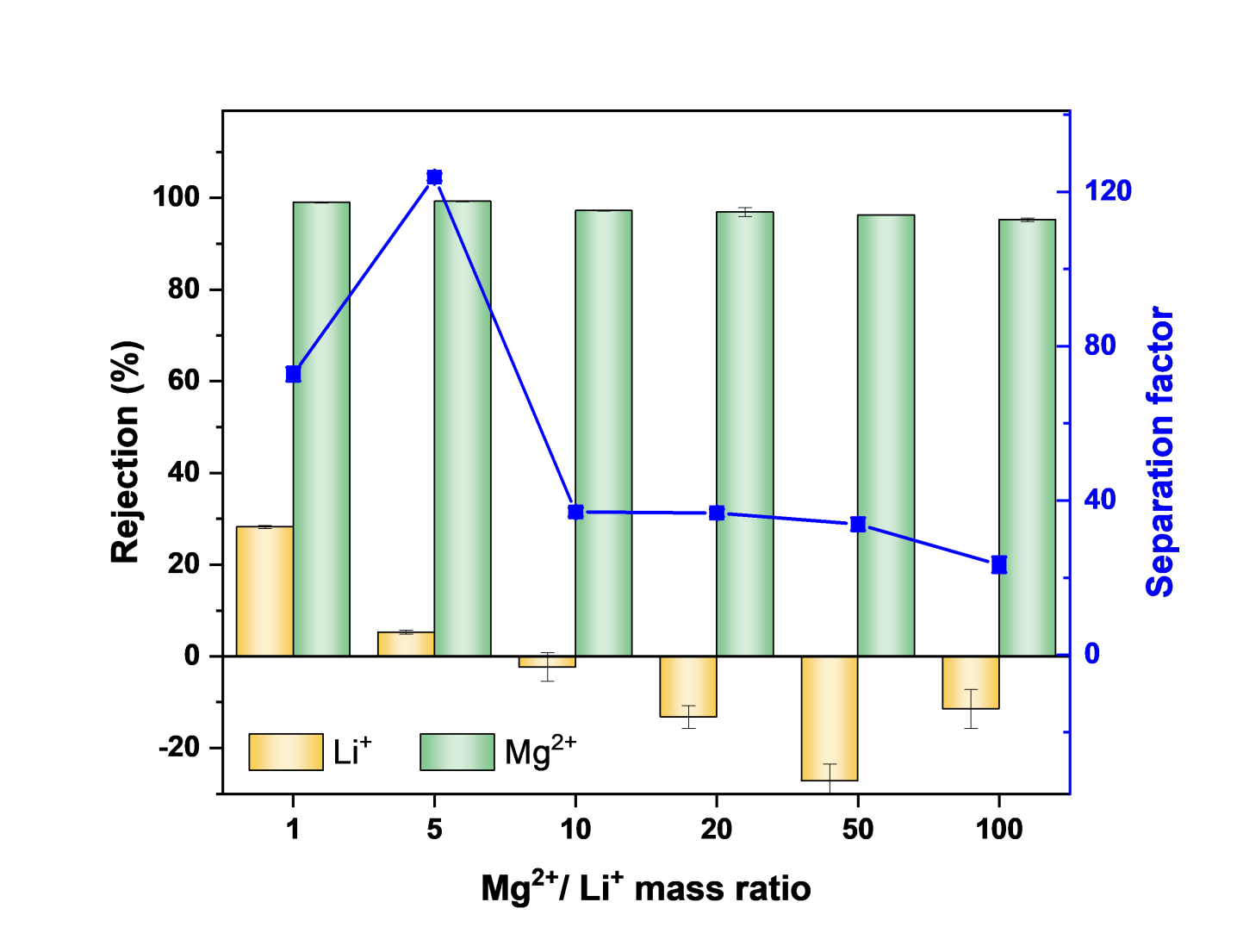


**Fig. S31** Rejection and separation factor of COF scaffold membranes in the mixed-solute system (MgSO_4_ and Li_2_SO_4_)


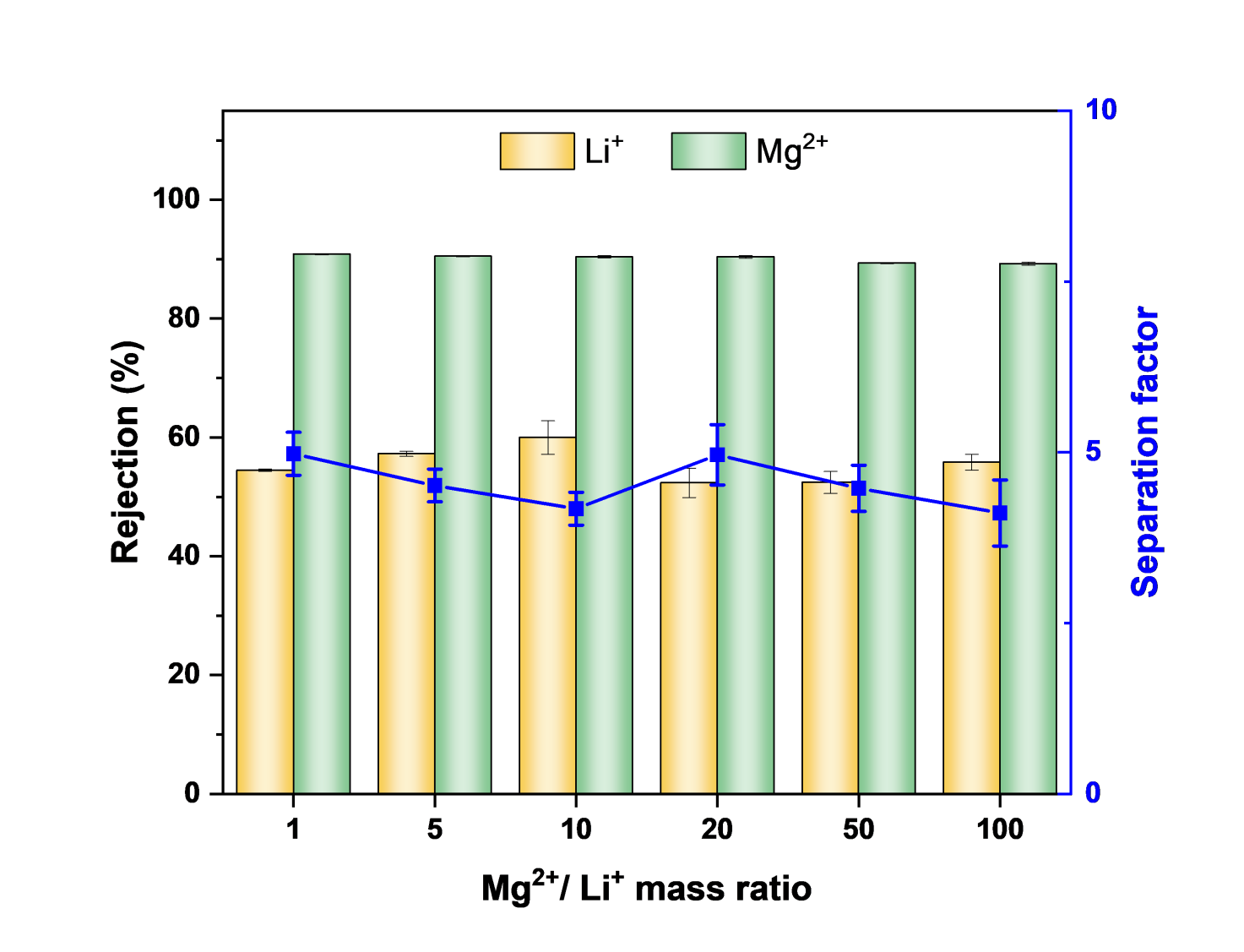


**Fig. S32** Rejection and separation factor of PU membranes in the single-solute system (MgSO_4_ and Li_2_SO_4_)


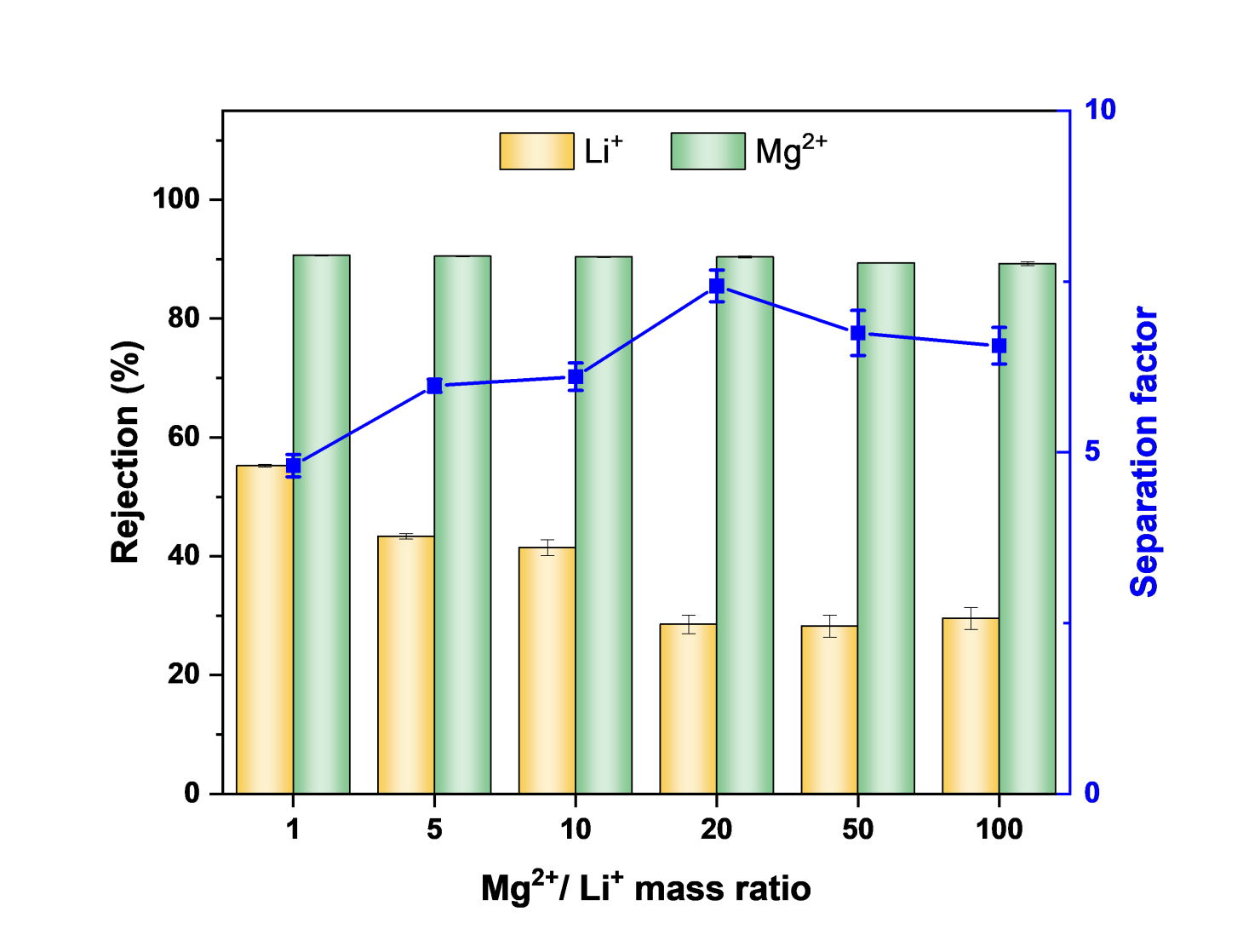


**Fig. S33** Rejection and separation factor of PU membranes in the mixed-solute system (MgSO_4_ and Li_2_SO_4_)


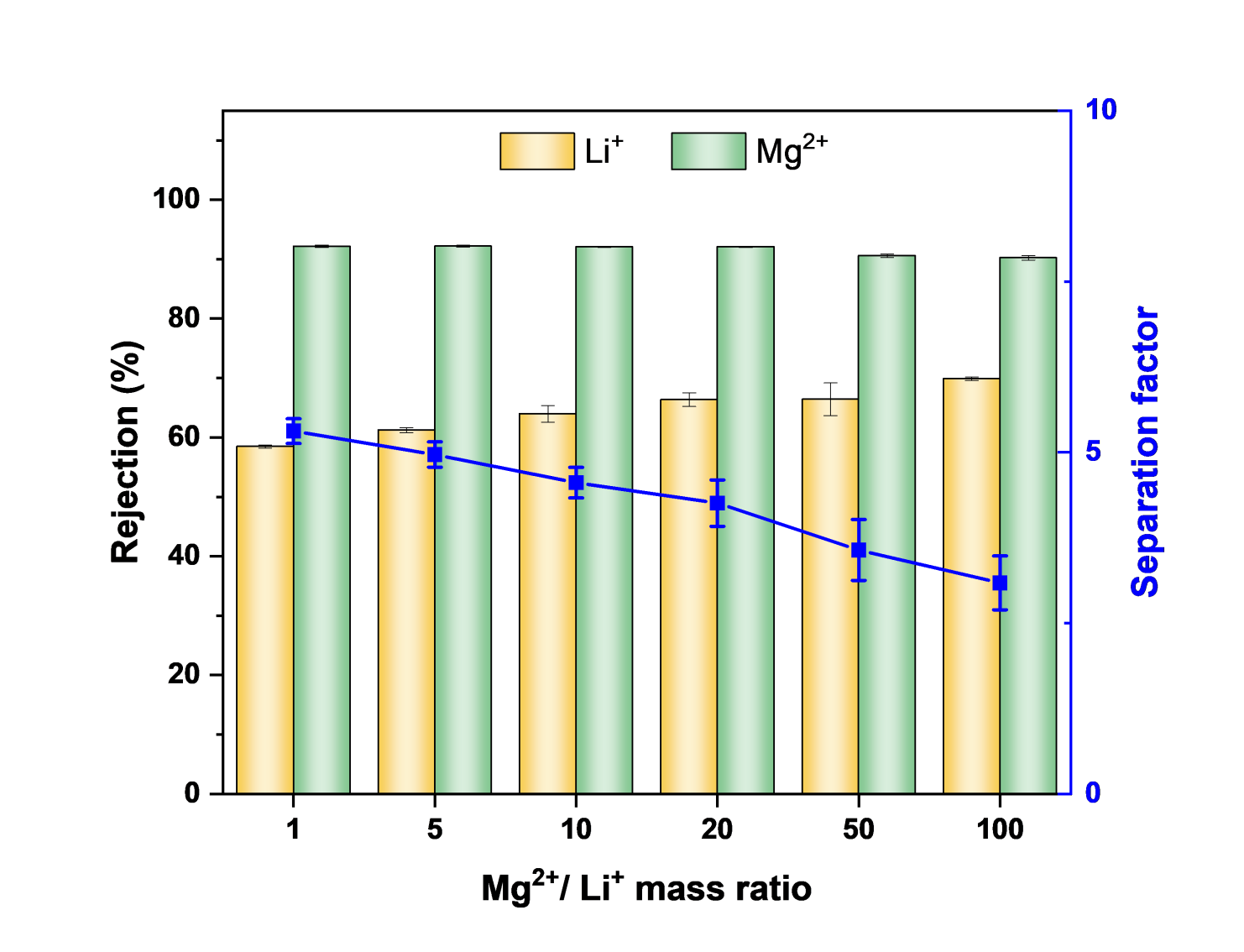


**Fig. S34** Rejection and separation factor of COF hybrid membranes in the single-solute system (MgSO_4_ and Li_2_SO_4_)


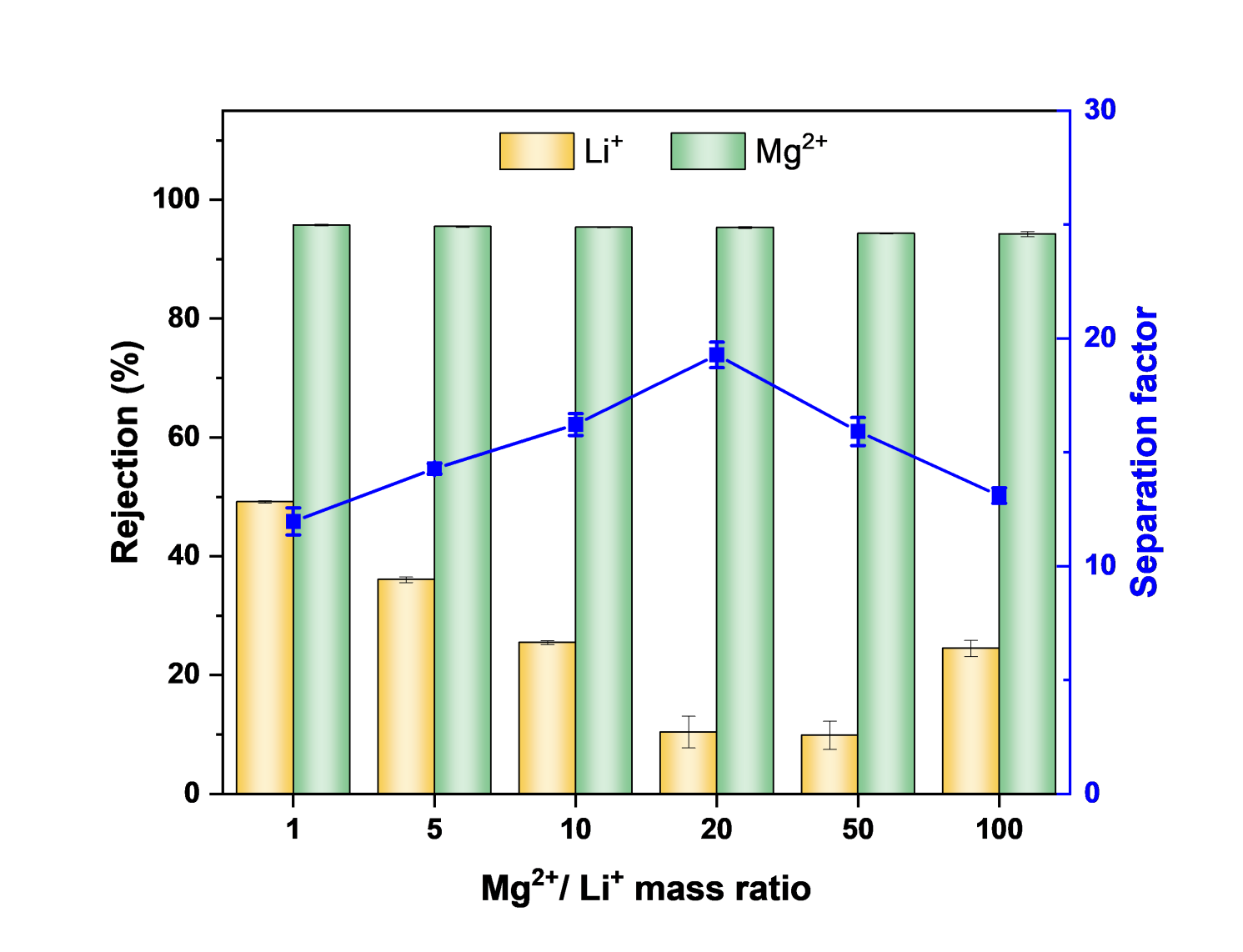


**Fig. S35** Rejection and separation factor of COF hybrid membranes in the mixed-solute system (MgSO_4_ and Li_2_SO_4_)


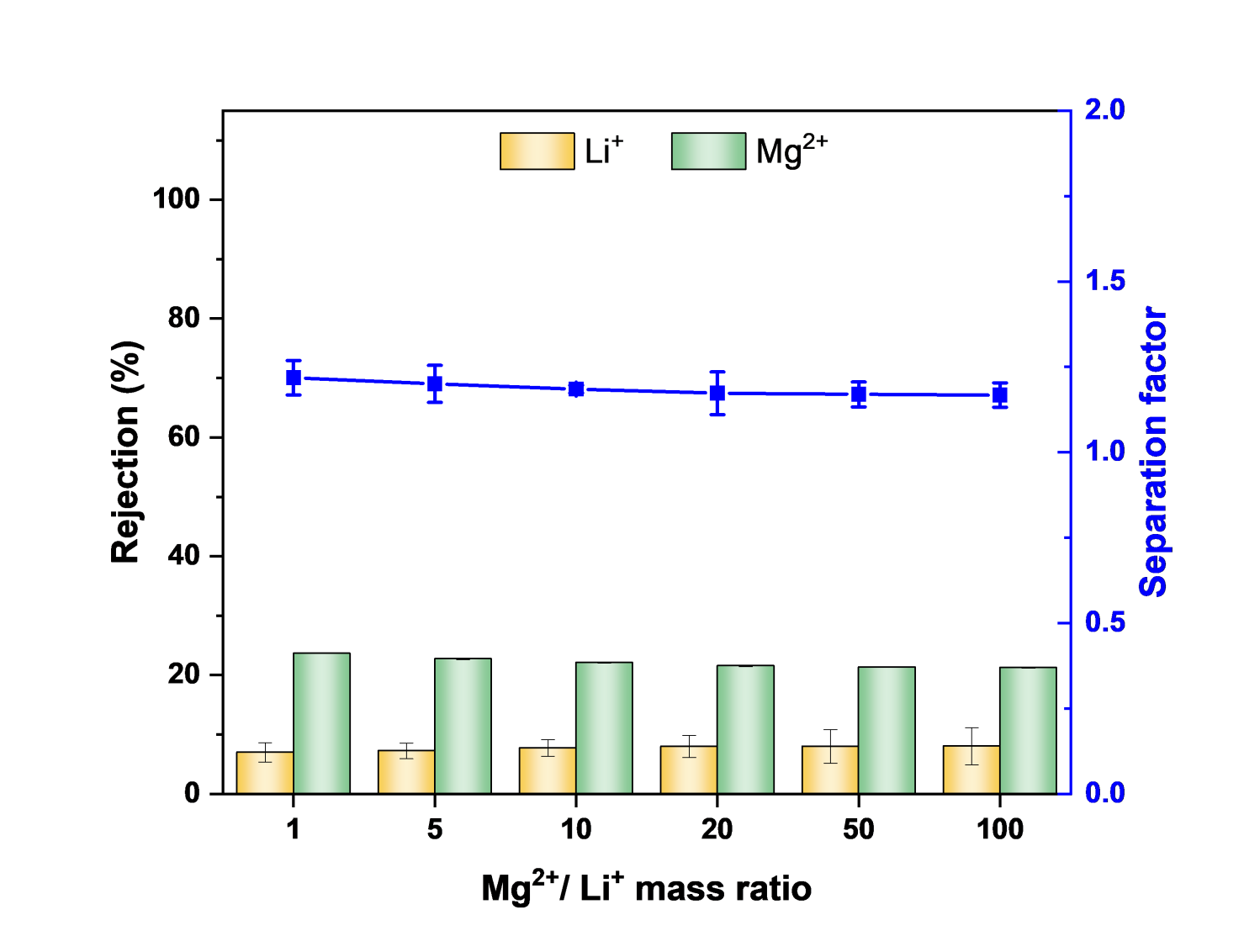


**Fig. S36** Rejection and separation factor of COF membranes in the single-solute system (MgSO_4_ and Li_2_SO_4_)


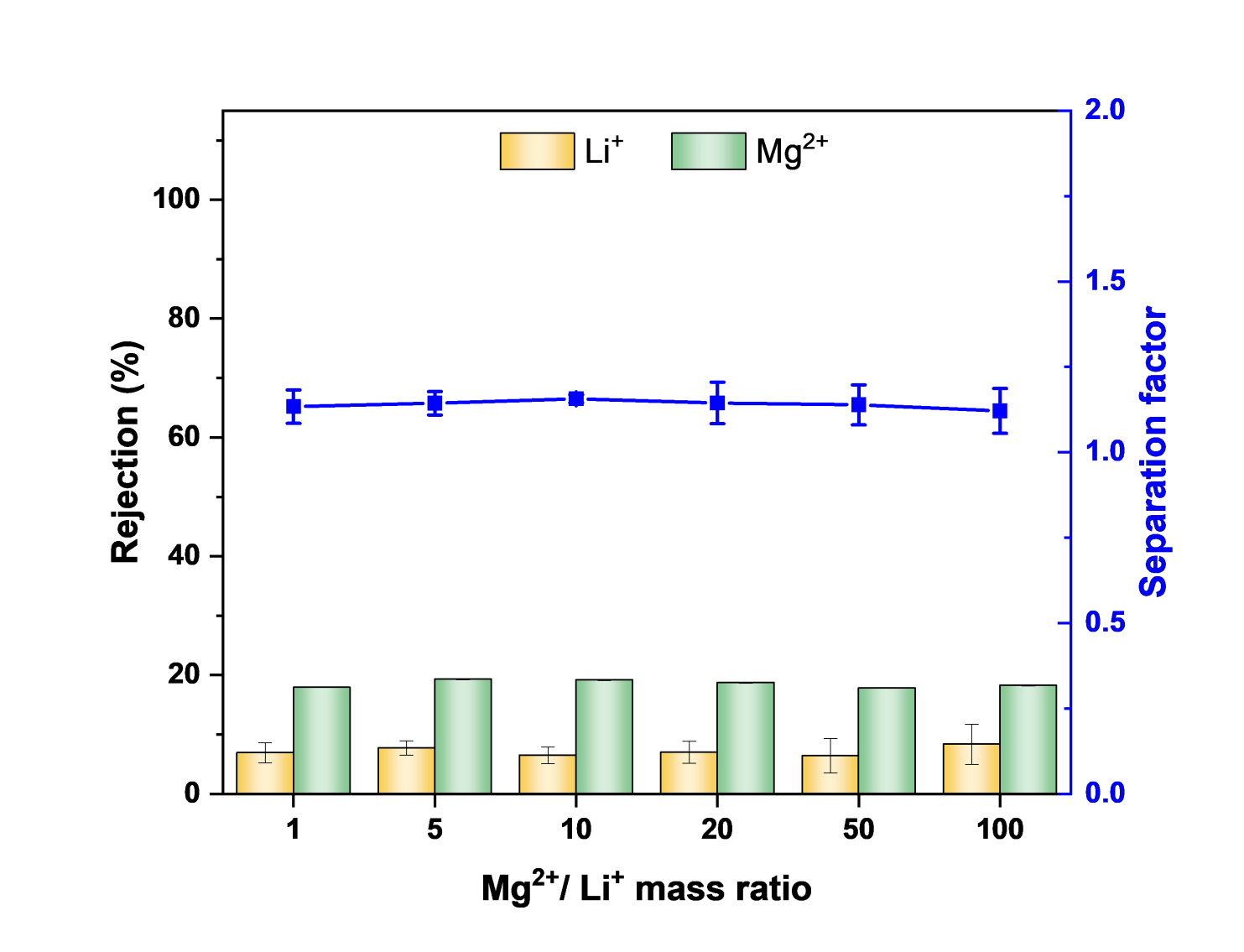


**Fig. S37** Rejection and separation factor of COF membranes in the mixed-solute system (MgSO_4_ and Li_2_SO_4_)

**Table S1** Mg^2+^/Li^+^ mass ratios in feed and permeate in the mixed-solute system

| Mg^2+^/Li^+^ mass ratio | |
| --- | --- |
| Feed | Permeate |
| 1 | 0.0066 |
| 5 | 0.0284 |
| 10 | 0.0511 |
| 20 | 0.1011 |
| 50 | 0.2156 |
| 100 | 1.7756 |

**Table S2** The summary of Li^+^/Mg^2+^ separation performance of mixed-solute system of nanofiltration membranes

| **Membrane** | **Permeance**  **(LMH/bar)** | **Mg^2+^/Li^+^ mass ratio** | **True selectivity** | **Testing conditions**  **(C_total_, Pressure)** | **References** |
| --- | --- | --- | --- | --- | --- |
| TFNPHF | 6.7 | 21.4 | 13.1 | 2000 ppm, 6 bar | **[S**8] |
| MBCN | 23 | 73 | 23.9 | 2000 ppm, 4 bar | **[S**9] |
| NF-HACC | 15.7 | 21.4 | 13.9 | 2000 ppm, 6 bar | [S10] |
| PEI-LDH/GA | 6.3 | 10 | 18.7 | 2000 ppm, 5 bar | [S11] |
| PEI/Cyclen-TMC | 14 | 20 | 8 | 2000 ppm, 5 bar | [S12] |
| UiO/TFN/PEI | 30.6 | 20 | 33 | 2000 ppm, 4 bar | [S13] |
| DTES/PEI/TMC | 6.2 | 20 | 12.95 | 2000 ppm, 8 bar | [S14] |
| SERS | 6.2 | 20 | 15.38 | 2000 ppm, 15 bar | [S15] |
| SIP | 1.3 | 20 | 7.68 | 2000 ppm, 15 bar |  |
| PIP-TMC/[MimAP][Tf_2_N] | 4.72 | 20 | 8.12 | 2100 ppm, 6 bar | [S16] |
|  | 4.72 | 40 | 6.19 | 4100 ppm, 6 bar |  |
| CA/PEI | 18.6 | 21.4 | 66.4 | 2000 ppm, 10 bar | [S17] |
| PAA/TMC | 7.39 | 20 | 82.8 | 2000 ppm, 5 bar | [S18] |
| PAH/DA/PSS | 21.9 | 35 | 37.8 | 2000 ppm, 2 bar | [S19] |
| NoriaPG/PEI | 22.5 | 30.9 | 88.6 | 2000 ppm, 10 bar | [S20] |
| Cu-MPD | 16.2 | 23 | 8 | 2000 ppm, 5 bar | [S21] |
| PGO | 6.85 | 23 | 23.5 | 5000 ppm, 2 bar | [S22] |
| PEI-TMC/DAIB | 15.5 | 15.3 | 16.6 | 5500 ppm, 6 bar | [S23] |
| PEI-TMC/QBPD | 26.11 | 50 | 5.2 | 2000 ppm, 6 bar | [S24] |
| PEI-TMC/QEDTP | 18.82 | 120 | 15.6 | 2000 ppm, 6 bar | [S25] |
| PEI-TMC/HMTAB | 16.32 | 50 | 10.1 | 2000 ppm, 6 bar | [S26] |
| PEI-TMC/TQAIL | 13.26 | 15.3 | 23.17 | 5500 ppm, 6 bar | [S27] |
| PEI-TMC/QTHIM | 33 | 50 | 10.9 | 2000 ppm, 6 bar | [S28] |
| PEI-g-PA | 12 | 20 | 33.4 | 2000 ppm, 4 bar | [S29] |
| PEI-TMC | 5.02 | 20 | 15.38 | 2000 ppm, 8 bar | [S30] |
| RIP | 22.25 | 20 | 9.22 | 2000 ppm, 15 bar | [S31] |
| polyamide-TG | 1.4 | 20 | 82.96 | 2000 ppm, 4 bar | [S32] |
| NF-TC | 8 | 20 | 167 | 2000 ppm, 5 bar | [S33] |
| COF scaffold membrane | 11.47 | 10 | 195.76 | 2000 ppm, 6 bar | This work |
|  |  | 20 | 197.75 |  |  |
|  |  | 50 | 231.87 |  |  |
|  |  | 100 | 56.32 |  |  |

**Table S3** The summary of reported diffusion coefficients

| **Membrane** | **Ion** | **Diffusion coefficient**  **(10^-5^ cm^2^ s^-1^)** | **References** |
| --- | --- | --- | --- |
| TpBDMe2 | K^+^ | 0.976 | [S34] |
|  | Na^+^ | 0.0865 |  |
|  | Li^+^ | 0.0632 |  |
|  | Mg^2+^ | 0.0185 |  |
| TpPa–CO_2_H | Rb^+^ | (5.7 ± 0.81) × 10^-4^ | [S35] |
|  | K^+^ | (7.6 ± 1.1) × 10^−4^ |  |
|  | Na^+^ | (6.3 ± 1.8) × 10^−4^ |  |
|  | Li^+^ | (4.2 ± 1.1) × 10^-4^ |  |
| COF-V-60% | K^+^ | 8.92 × 10^-7^ | [S36] |
|  | Na^+^ | 5.25 × 10^-7^ |  |
| TpTag-COF | Cl^-^ | 0.1524 ± 0.0501 | [S37] |
| COF-170/PAN | Cl^-^ | 0.815 | [S38] |
|  | SO_4_^2−^ | 0.477 |  |
| COF scaffold membrane | Li^+^  Cl^-^ | 0.989  2.141 | This work |
|  |  |  |  |

**Supplementary References**

1. Y. Kong, B. Lyu, C. Fan, Y. Yang, X. Wang et al., Manipulation of cationic group density in covalent organic framework membranes for efficient anion transport. J. Am. Chem. Soc. **145**(51), 27984–27992 (2023). <https://doi.org/10.1021/jacs.3c07958>
2. D. Van Der Spoel, E. Lindahl, B. Hess, G. Groenhof, A.E. Mark et al., GROMACS: Fast, flexible, and free. J. Comput. Chem. **26**(16), 1701–1718 (2005). <https://doi.org/10.1002/jcc.20291>
3. W.L. Jorgensen, J. Chandrasekhar, J.D. Madura, R.W. Impey, M.L. Klein, Comparison of simple potential functions for simulating liquid water. J. Chem. Phys. **79**(2), 926–935 (1983). <https://doi.org/10.1063/1.445869>
4. T. Darden, D. York, L. Pedersen, Particle mesh Ewald: an *N*⋅log(*N*) method for Ewald sums in large systems. J. Chem. Phys. **98**(12), 10089–10092 (1993). <https://doi.org/10.1063/1.464397>
5. B. Hess, H. Bekker, H.J.C. Berendsen, J.G.E.M. Fraaije, LINCS: a linear constraint solver for molecular simulations. J. Comput. Chem. **18**(12), 1463–1472 (1997). <https://doi.org/10.1002/(SICI)1096-987X(199709)18:12&lt;1463::AID-JCC4&gt;3.0.CO;2-H>
6. H.J.C. Berendsen, J.P.M. Postma, W.F. van Gunsteren, A. DiNola, J.R. Haak, Molecular dynamics with coupling to an external bath. J. Chem. Phys. **81**(8), 3684–3690 (1984). <https://doi.org/10.1063/1.448118>
7. R. Martoňák, A. Laio, M. Parrinello, Predicting crystal structures: the parrinello-rahman method revisited. Phys. Rev. Lett. **90**(7), 075503 (2003). <https://doi.org/10.1103/physrevlett.90.075503>
8. Q. Shen, S.-J. Xu, Z.-L. Xu, H.-Z. Zhang, Z.-Q. Dong, Novel thin-film nanocomposite membrane with water-soluble polyhydroxylated fullerene for the separation of Mg^2+^/Li^+^ aqueous solution. J. Appl. Polym. Sci. **136**(41), 48029 (2019). <https://doi.org/10.1002/app.48029>
9. Q. Bi, C. Zhang, J. Liu, X. Liu, S. Xu, Positively charged zwitterion-carbon nitride functionalized nanofiltration membranes with excellent separation performance of Mg^2+^/Li^+^ and good antifouling properties. Sep. Purif. Technol. **257**, 117959 (2021). <https://doi.org/10.1016/j.seppur.2020.117959>
10. T. Zhang, Y. Chen, Q. Yu, H. Sun, K. Chen et al., Advanced Mg^2+^/Li^+^ separation nanofiltration membranes by introducing hydroxypropyltrimethyl ammonium chloride chitosan as a co-monomer. Appl. Surf. Sci. **616**, 156434 (2023). <https://doi.org/10.1016/j.apsusc.2023.156434>
11. H. Ni, N. Wang, Y. Yang, M. Shen, Q.-F. An, Positively-charged nanofiltration membrane constructed by polyethyleneimine/layered double hydroxide for Mg^2+^/Li^+^ separation. Desalination **548**, 116256 (2023). <https://doi.org/10.1016/j.desal.2022.116256>
12. T. Li, X. Zhang, Y. Zhang, J. Wang, Z. Wang et al., Nanofiltration membrane comprising structural regulator Cyclen for efficient Li^+^/Mg^2+^ separation. Desalination **556**, 116575 (2023). <https://doi.org/10.1016/j.desal.2023.116575>
13. F. Aghili, A.A. Ghoreyshi, B. Van der Bruggen, A. Rahimpour, A highly permeable UiO-66-NH2/polyethyleneimine thin-film nanocomposite membrane for recovery of valuable metal ions from brackish water. Process. Saf. Environ. Prot. **151**, 244–256 (2021). <https://doi.org/10.1016/j.psep.2021.05.022>
14. H. Wu, H. Zhao, Y. Lin, X. Liu, L. Wang et al., Positively-charged PEI/TMC nanofiltration membrane prepared by adding a diamino-silane coupling agent for Li^+^/Mg^2+^ separation. J. Membr. Sci. **672**, 121468 (2023). <https://doi.org/10.1016/j.memsci.2023.121468>
15. Y. Li, S. Wang, H. Li, D. Liu, Y. Jin et al., Polyamide nanofiltration membranes with rigid–flexible microstructures for high-efficiency Mg^2+^/Li^+^ separation. Sep. Purif. Technol. **306**, 122552 (2023). <https://doi.org/10.1016/j.seppur.2022.122552>
16. H. Wu, Y. Lin, W. Feng, T. Liu, L. Wang et al., A novel nanofiltration membrane with [MimAP] [Tf2N] ionic liquid for utilization of lithium from brines with high Mg^2+^/Li^+^ ratio. J. Membr. Sci. **603**, 117997 (2020). <https://doi.org/10.1016/j.memsci.2020.117997>
17. K. Chen, S. Zhao, H. Lan, T. Xie, H. Wang et al., Dual-electric layer nanofiltration membranes based on polyphenol/PEI interlayer for highly efficient Mg^2+^/Li^+^ separation. J. Membr. Sci. **660**, 120860 (2022). <https://doi.org/10.1016/j.memsci.2022.120860>
18. P. Xu, R.R. Gonzales, J. Hong, K. Guan, Y.-H. Chiao et al., Fabrication of highly positively charged nanofiltration membranes by novel interfacial polymerization: Accelerating Mg^2+^ removal and Li^+^ enrichment. J. Membr. Sci. **668**, 121251 (2023). <https://doi.org/10.1016/j.memsci.2022.121251>
19. Y. Yang, Y. Li, K. Goh, C.H. Tan, R. Wang, Dopamine-intercalated polyelectrolyte multilayered nanofiltration membranes: Toward high permselectivity and ion-ion selectivity. J. Membr. Sci. **648**, 120337 (2022). <https://doi.org/10.1016/j.memsci.2022.120337>
20. K. Chen, F. Li, T. Wei, H. Zhou, T. Zhang et al., An interlayer-based positive charge compensation strategy for the preparation of highly selective Mg^2+^/Li^+^ separation nanofiltration membranes. J. Membr. Sci. **684**, 121882 (2023). <https://doi.org/10.1016/j.memsci.2023.121882>
21. L. Wang, D. Rehman, P.-F. Sun, A. Deshmukh, L. Zhang et al., Novel positively charged metal-coordinated nanofiltration membrane for lithium recovery. ACS Appl. Mater. Interfaces **13**(14), 16906–16915 (2021). <https://doi.org/10.1021/acsami.1c02252>
22. R. Wang, J. Wu, J. Zheng, B. Chen, X. Zhu, Janus membrane with tailored upper and lower surface charges for ion penetration manipulation in high-performance nanofiltration. J. Membr. Sci. **667**, 121191 (2023). <https://doi.org/10.1016/j.memsci.2022.121191>
23. H. Peng, Q. Zhao, A nano-heterogeneous membrane for efficient separation of lithium from high magnesium/lithium ratio brine. Adv. Funct. Mater. **31**(14), 2009430 (2021). <https://doi.org/10.1002/adfm.202009430>
24. Y. Feng, H. Peng, Q. Zhao, Fabrication of high performance Mg^2+^/Li^+^ nanofiltration membranes by surface grafting of quaternized bipyridine. Sep. Purif. Technol. **280**, 119848 (2022). <https://doi.org/10.1016/j.seppur.2021.119848>
25. Y. Xu, H. Peng, H. Luo, Q. Zhang, Z. Liu et al., High performance Mg^2+^/Li^+^ separation membranes modified by a bis-quaternary ammonium salt. Desalination **526**, 115519 (2022). <https://doi.org/10.1016/j.desal.2021.115519>
26. H. Luo, H. Peng, Q. Zhao, High flux Mg^2+^/Li^+^ nanofiltration membranes prepared by surface modification of polyethylenimine thin film composite membranes. Appl. Surf. Sci. **579**, 152161 (2022). <https://doi.org/10.1016/j.apsusc.2021.152161>
27. F. Soyekwo, H. Wen, D. Liao, C. Liu, Fouling-resistant ionic graft-polyamide nanofiltration membrane with improved permeance for lithium separation from MgCl_2_/LiCl mixtures. J. Membr. Sci. **659**, 120773 (2022). <https://doi.org/10.1016/j.memsci.2022.120773>
28. X. Liu, Y. Feng, Y. Ni, H. Peng, S. Li et al., High-permeance Mg^2+^/Li^+^ separation nanofiltration membranes intensified by quadruple imidazolium salts. J. Membr. Sci. **667**, 121178 (2023). <https://doi.org/10.1016/j.memsci.2022.121178>
29. Z. Yang, W. Fang, Z. Wang, R. Zhang, Y. Zhu et al., Dual-skin layer nanofiltration membranes for highly selective Li^+^/Mg^2+^ separation. J. Membr. Sci. **620**, 118862 (2021). <https://doi.org/10.1016/j.memsci.2020.118862>
30. P. Xu, W. Wang, X. Qian, H. Wang, C. Guo et al., Positive charged PEI-TMC composite nanofiltration membrane for separation of Li^+^ and Mg^2+^ from brine with high Mg^2+^/Li^+^ ratio. Desalination **449**, 57–68 (2019). <https://doi.org/10.1016/j.desal.2018.10.019>
31. Y. Li, S. Wang, W. Wu, H. Yu, R. Che et al., Fabrication of positively charged nanofiltration membrane with uniform charge distribution by reversed interfacial polymerization for Mg^2+^/Li^+^ separation. J. Membr. Sci. **659**, 120809 (2022). <https://doi.org/10.1016/j.memsci.2022.120809>
32. S. Zhang, R. Zhang, R. Li, Z. Zhang, Y. Li et al., Guanidyl-incorporated nanofiltration membranes toward superior Li^+^/Mg^2+^ selectivity under weakly alkaline environment. J. Membr. Sci. **663**, 121063 (2022). <https://doi.org/10.1016/j.memsci.2022.121063>
33. G. Zhao, Y. Zhang, Y. Li, G. Pan, Y. Liu, Positively charged nanofiltration membranes for efficient Mg^2+^/Li^+^ separation from high Mg^2+^/Li^+^ ratio brine. Adv. Membr. **3**, 100065 (2023). <https://doi.org/10.1016/j.advmem.2023.100065>
34. F. Sheng, B. Wu, X. Li, T. Xu, M.A. Shehzad et al., Efficient ion sieving in covalent organic framework membranes with sub-2-nanometer channels. Adv. Mater. **33**(44), 2104404 (2021). <https://doi.org/10.1002/adma.202104404>
35. H. Wang, Y. Zhai, Y. Li, Y. Cao, B. Shi et al., Covalent organic framework membranes for efficient separation of monovalent cations. Nat. Commun. **13**, 7123 (2022). <https://doi.org/10.1038/s41467-022-34849-7>
36. L. Cao, I.-C. Chen, Z. Li, X. Liu, M. Mubashir et al., Switchable Na(+) and K(+) selectivity in an amino acid functionalized 2D covalent organic framework membrane. Nat. Commun. **13**(1), 7894 (2022). <https://doi.org/10.1038/s41467-022-35594-7>
37. Q.-W. Meng, S. Wu, M. Liu, Q. Guo, W. Xian et al., Guanidinium-based covalent organic framework membrane for single-acid recovery. Sci. Adv. **9**(25), eadh0207 (2023). <https://doi.org/10.1126/sciadv.adh0207>
38. Q.-W. Meng, J. Li, Z. Lai, W. Xian, S. Wang et al., Optimizing selectivity *via* membrane molecular packing manipulation for simultaneous cation and anion screening. Sci. Adv. **10**(39), eado8658 (2024). <https://doi.org/10.1126/sciadv.ado8658>
